# A single glucocorticoid response element regulates sociability in a sex-specific manner

**DOI:** 10.1038/s41380-025-03158-y

**Published:** 2025-08-25

**Authors:** Brian F. Corbett, Jay Arner, Sandra Luz, Jason Yan, Jose Castro-Vildosola, Tamara Hala, Deanne Taylor, Seema Bhatnagar

**Affiliations:** 1 Center for Stress Neurobiology, Children’s Hospital of Philadelphia, Philadelphia, PA USA; 2 Department of Biomedical and Health Informatics, Children’s Hospital of Philadelphia, Philadelphia, PA USA; 3 The Perelman School of Medicine, University of Pennsylvania, Philadelphia, PA USA; 4 Present Address: Department of Biology, Rutgers, The State University of New Jersey, Camden, NJ USA; 5 Present Address: Center for Computational and Integrative Biology, Camden, NJ USA

**Keywords:** Neuroscience, Physiology

## Abstract

Glucocorticoid receptors (GRs) regulate transcription to reduce inflammatory processes, modulate neuron function, and influence behavior. However, the precise loci bound by GRs that are critically important for these effects have not been fully determined. Here, we deleted the GR binding site near the sphingosine-1-phosphate receptor 3 (S1PR3) gene using a CRISPR/Cas9 approach in rats (S1PR3^GRE-/GRE-^ rats). Socially defeated S1PR3^GRE-/GRE-^ males displayed increased inflammatory markers and reduced sociability compared to defeated wild-type (WT) controls. Similar effects were observed in non-stressed females, indicating a greater dependence on the regulation of S1PR3 by GRs in females. Coherent neural activity between the locus coeruleus (LC) and medial prefrontal cortex (mPFC) was increased in defeated S1PR3^GRE-/GRE-^ males whereas increases were observed in non-stressed S1PR3^GRE-/GRE^ females. Chemogenetically inhibiting mPFC-projecting LC neurons during defeat increased subsequent social interaction in WT and S1PR3^GRE-/GRE-^ males. Together, these findings demonstrate that GR-induced S1PR3 mitigates inflammatory processes and promotes resilience by reducing coherent neural activity between the LC and mPFC and may be an important mechanism through which the effects of stress in females can be mitigated.

## Introduction

Stress contributes to the onset and development of mood-related disorders like generalized anxiety, major depressive disorder (MDD), and post-traumatic stress disorder (PTSD) [1–3]. While lifetime exposure to traumatic stress is at least 40% in Western countries, the prevalence of these disorders is lower [4–9]. Therefore, certain individuals are more vulnerable to the adverse effects of stress whereas others are more resilient. Multiple biological factors contribute to stress vulnerability, including inflammatory processes. Stress-related disorders share comorbidity with immune disorders [10–13]. Inflammatory markers, like increased neutrophil-to-lymphocyte ratio [14, 15] and elevated cytokine levels in the blood [16–20] and brain [21], are hallmarks of stress-related disorders like PTSD [22] and MDD [15, 23]. Cytokine-based treatments for hepatitis and cancer increase symptoms of depression [24–26]. Conversely, anti-inflammatory treatments improve depression scores [27], especially in depressed patients displaying increased inflammatory markers in blood [28]. Therefore, stress increases inflammatory processes that contribute to the onset and development of stress-related disorders.

Glucocorticoids like corticosterone (rodents) and cortisol (humans) counter inflammatory processes [29, 30] that can be increased by noradrenergic neurotransmission during stress [31, 32]. Concentrations of circulating glucocorticoids are increased by activation of the hypothalamic-pituitary-adrenal (HPA) axis [33]. Glucocorticoids bind to glucocorticoid receptors (GRs) in peripheral tissues and the brain [34, 35]. Once bound to their ligand, GRs translocate to the nucleus and bind to specific DNA sequences called glucocorticoid response elements (GREs). GRs modulate the expression of genes that regulate a wide range of biological processes [36], including reducing inflammatory gene expression [37]. When the anti-inflammatory effects of GRs are impaired, the pro-inflammatory effects of stress can go unchecked, leading to systemic increases in inflammatory markers [30].

We previously demonstrated that GR densities are increased in the medial prefrontal cortex (mPFC) of rats exposed to chronic social defeat, in which rats are challenged with exposure to a conspecific aggressor. Subpopulations of rats actively cope during stress by displaying longer defeat latencies. These rats do not exhibit changes in sociability or depression-like behavior following social defeat stress and are considered resilient to stress. In contrast, another subpopulation, considered vulnerable, displays passive coping, as assessed by short defeat latencies. Following defeat, vulnerable rats display depression-like behavior and reduced sociability [38–40]. We showed that GR-induced sphingosine-1-phosphate receptor 3 (S1PR3, endothelial differentiation gene-3) expression is elevated in the mPFC of resilient rats and promotes active coping and sociability by mitigating stress-induced inflammatory processes in the mPFC. Social defeat stress also increased S1PR3 mRNA in the blood of resilient, but not vulnerable, rats. Our findings were translationally relevant: blood S1PR3 mRNA is reduced in veterans with PTSD compared to combat-exposed controls and inversely correlates with PTSD symptom severity [38]. Here, in order to further study the consequences of GR regulation of the S1PR3, we used a CRISPR/Cas9-based approach to delete the GRE upstream of the transcriptional start site for S1PR3 in male and female rats (S1PR3^GRE-/GRE-^ rats). Overall, our findings show that deletion of the GRE near S1PR3 plays an important role in mitigating the adverse effects of stress.

## GRs increase S1PR3 expression in females and defeated males and increase sociability

**Fig. 1:**
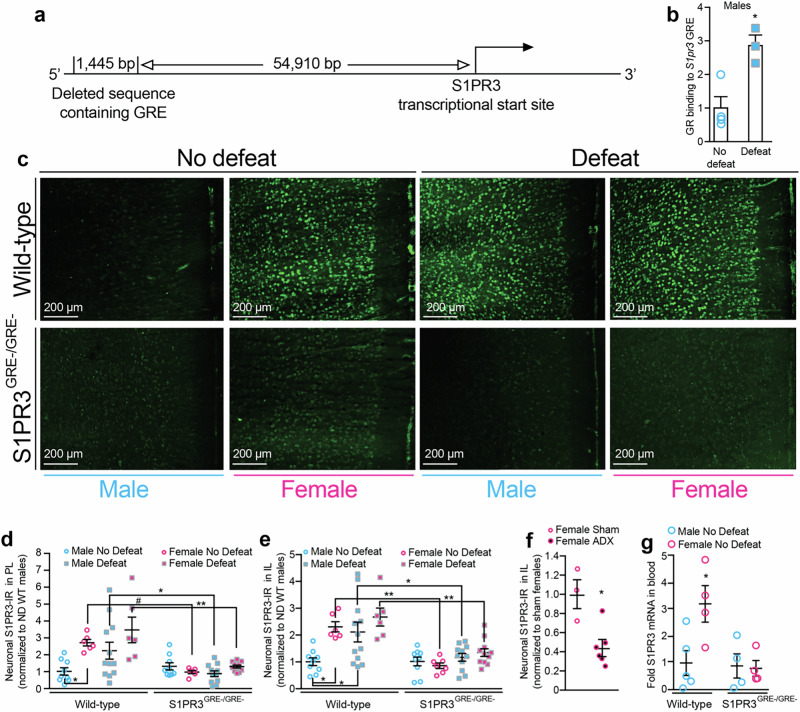
GR-induced S1PR3 is impaired in S1PR3^GRE-/GRE-^ rats. **a** Graphic model of deleted GRE in S1PR3^GRE-/GRE-^ rats. **b** GR binding to the GRE upstream of the S1PR3 gene in non-defeated (*n* = 3) and defeated (*n* = 4) male WT rats. **c** Images and quantification of S1PR3 in the **d** PL and **e** IL of non-defeated WT males (*n* = 9), non-defeated WT females (*n* = 6), defeated WT males (*n* = 12), defeated WT females (*n* = 6), non-defeated S1PR3^GRE-/GRE-^ males (*n* = 9), non-defeated S1PR3^GRE-/GRE-^ females (*n* = 7), defeated S1PR3^GRE-/GRE-^ males (*n* = 11), and defeated S1PR3^GRE-/GRE-^ females (*n* = 11). For d, genotype effect *p* < 0.0001, defeat effect *p* = 0.06, sex effect *p* = 0.0041. For e, genotype effect *p* < 0.0001, defeat effect *p* = 0.0039, sex effect *p* = 0.0098. **f** S1PR3 protein in the IL of sham control (*n* = 3) and adrenalectomized (ADX, *n* = 6) non-defeated females. **g** S1PR3 mRNA in whole blood taken from non-defeated WT males (*n* = 5), WT females (*n* = 4), S1PR3^GRE-/GRE-^ males (*n* = 4), and S1PR3^GRE-/GRE-^ females (*n* = 4). Lines represent means ± SEM. For **b** and **f**, *p < 0.05; unpaired two-tailed Student’s t-test. For **d** and **e**, *p < 0.05, **p < 0.01, #*p* < 0.10; Tukey’s multiple comparisons following 3-way ANOVA. For **g**, *p < 0.05 compared to all other groups; Tukey’s multiple comparisons following 2-way ANOVA.

We previously demonstrated that GR knockdown reduces S1PR3 expression in the mPFC of stressed male rats [38]. We hypothesized that the GRE upstream of the S1PR3 gene is necessary for GR-induced S1PR3 expression. In mice, a GRE has been identified near the S1PR3 gene, but not near other genes encoding S1PRs [41]. Using a BLAST-like alignment tool (BLAT), we identified the homologous sequence in rats, which is 54,910 bp upstream from the transcriptional start site for S1PR3 (Fig. 1a). GRs bind to this sequence in the mPFC as GR binding was increased at this locus in defeated WT males compared to non-defeated males (Fig. 1b). No amplification of this deleted locus was observed for S1PR3^GRE-/GRE-^ rats. Male and female WT and S1PR3^GRE-/GRE-^ rats were either subjected to seven days of social defeat or served as novel cage controls. In WT rats, S1PR3 levels were increased in the prelimbic (PL) and infralimbic (IL) cortices of non-defeated females compared to non-defeated males. Differences in S1PR3 were not observed in male and female S1PR3^GRE-/GRE-^ rats. S1PR3 was lower in female S1PR3^GRE-/GRE-^ rats compared to female WT rats regardless of defeat condition. Consistent with our previous findings [38], defeat increased S1PR3 in the IL of male WT rats. Male S1PR3^GRE-/GRE-^ rats did not display increases in S1PR3 following defeat (Fig. 1c–e). We hypothesized that increased S1PR3 in non-defeated WT, but not S1PR3^GRE-/GRE-^, females is higher compared to non-defeated WT males because baseline plasma corticosterone concentrations are 5–10× higher in female rats compared to males [42, 43]. We performed either a sham control or bilateral adrenalectomy (ADX) on wild-type female rats and quantified S1PR3 in the IL seven days later. S1PR3 in the IL was reduced in ADX females compared to females receiving sham surgery (Fig. 1f, Supplementary Fig. 1a). ADX reduced plasma corticosterone concentrations 75-fold (Supplementary Fig. 1b). We found that in the absence of defeat, S1PR3 mRNA in whole blood was increased in WT, but not S1PR3^GRE-/GRE-^, females compared to WT males (Fig. 1g). In defeated males, S1PR3 mRNA was reduced in S1PR3^GRE-/GRE-^ rats compared to defeated WT littermates (Supplementary Fig. 1c). Together, these findings show that corticosterone-activated GRs increase S1PR3 expression in the mPFC and blood. Compared to non-defeated WT males, the higher levels of S1PR3 in defeated WT males and non-defeated WT females can be explained by increased levels of circulating corticosterone.

**Fig. 2:**
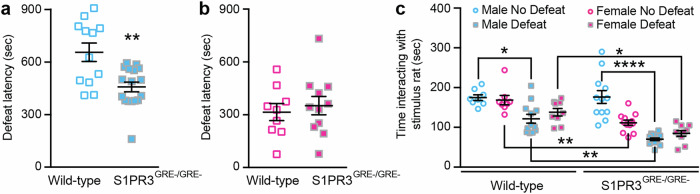
Social interaction is reduced in defeated S1PR3^GRE-/GRE-^ males and non-defeated S1PR3^GRE-/GRE-^ females. Defeat latencies are reduced in **a** S1PR3^GRE-/GRE-^ males (*n* = 16) compared to WT males (*n* = 12) but not in **b** S1PR3^GRE-/GRE-^ females (*n* = 11) compared to WT females (*n* = 9). **c** Time interacting with the stimulus rat in the social interaction paradigm in non-defeated WT males (*n* = 8), non-defeated WT females (*n* = 8), defeated WT males (*n* = 12), defeated WT females (*n* = 9), non-defeated S1PR3^GRE-/GRE-^ males (*n* = 12), non-defeated S1PR3^GRE-/GRE-^ females (*n* = 12), defeated S1PR3^GRE-/GRE-^ males (*n* = 16), and defeated S1PR3^GRE-/GRE-^ females (*n* = 10). Genotype effect *p* < 0.0001, defeat effect *p* < 0.0001. Lines represent means ± SEM. For **a**, ^**^p < 0.01; unpaired two-tailed Student’s t-test. For **c**, ^*^p < 0.05, ^**^p < 0.01, ^****^p < 0.0001; Tukey’s multiple comparisons following 3-way ANOVA.

We previously demonstrated that S1PR3 knockdown in the mPFC reduces defeat latencies (reduces active coping) in the resident-intruder paradigm and reduces social interaction, indicating a less resilient phenotype [38]. Defeat latency is reduced in male S1PR3^GRE-/GRE-^ rats compared to WT littermates (Fig. 2a). This effect was not observed in females (Fig. 2b) using a resident-intruder paradigm in which a lactating female is used as an aggressor. However, social defeat reduced social interaction in both WT males and females, an effect that was exacerbated in S1PR3^GRE-/GRE-^ rats. Social interaction was reduced in S1PR3^GRE-/GRE-^ females compared to WT female litter mates even in the absence of stress (Fig. 2c). Together, these findings indicate that GR-induced S1PR3 expression is necessary for active coping and sociability in defeated males and promotes social interaction during both baseline and stressed conditions in females.

## Central and peripheral inflammatory processes are increased in S1PR3^GRE-/GRE-^ rats

**Fig. 3:**
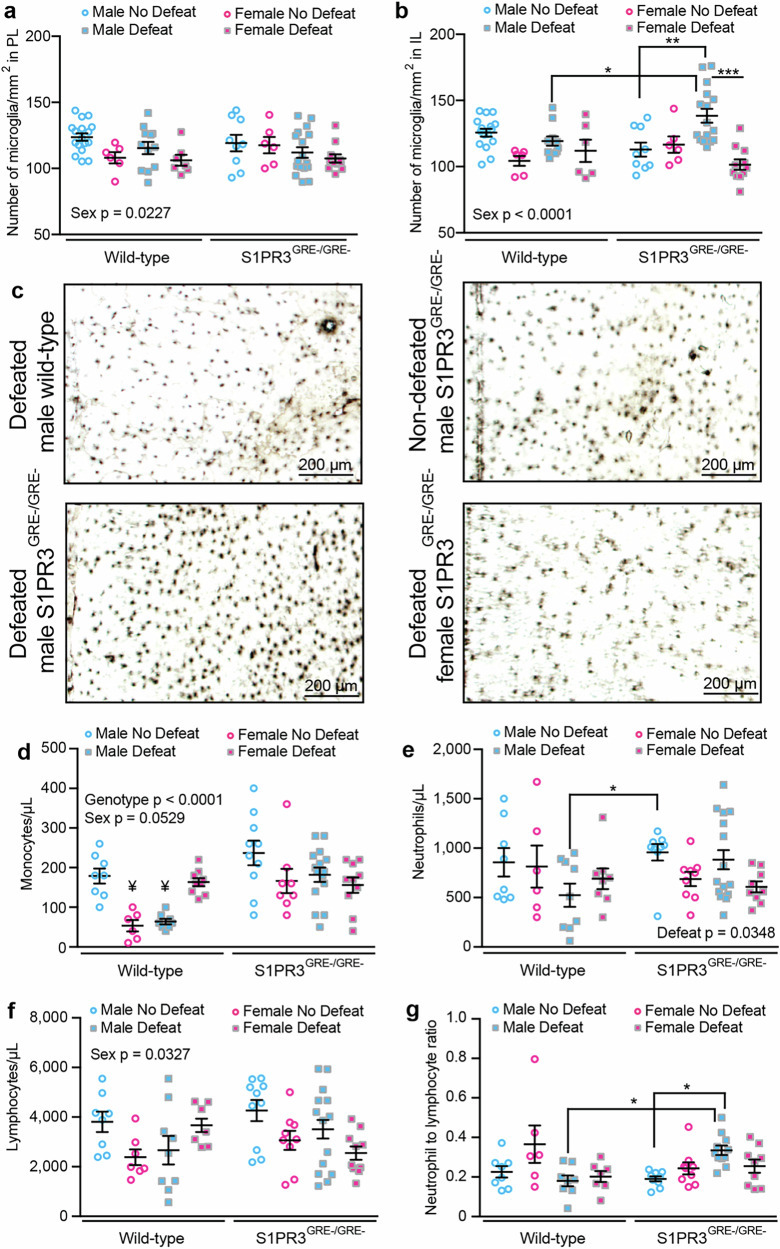
Inflammatory processes are increased in S1PR3^GRE-/GRE-^ rats. IBA+ cells in the **a** PL and **b** IL of non-defeated WT males (*n* = 16), non-defeated WT females (*n* = 6), defeated WT males (*n* = 12), defeated WT females (*n* = 6), non-defeated S1PR3^GRE-/GRE-^ males (*n* = 9), non-defeated S1PR3^GRE-/GRE-^ females (*n* = 6), defeated S1PR3^GRE-/GRE-^ males (*n* = 16), and defeated S1PR3^GRE-/GRE-^ females (*n* = 11). **c** Images of IBA1+ cells in the IL. **d** Monocyte concentrations from non-defeated WT males (*n* = 8), non-defeated WT females (*n* = 6), defeated WT males (*n* = 8), defeated WT females (*n* = 9), non-defeated S1PR3^GRE-/GRE-^ males (*n* = 10), non-defeated S1PR3^GRE-/GRE-^ females (*n* = 8), defeated S1PR3^GRE-/GRE-^ males (*n* = 15), and defeated S1PR3^GRE-/GRE-^ females (*n* = 10). Concentrations of **e** neutrophils, **f** lymphocytes, and **g** the neutrophil-to-lymphocyte ratio in non-defeated WT males (*n* = 8), non-defeated WT females (*n* = 6), defeated WT males (*n* = 9), defeated WT females (*n* = 7), non-defeated S1PR3^GRE-/GRE-^ males (*n* = 10), non-defeated S1PR3^GRE-/GRE-^ females (*n* = 8), defeated S1PR3^GRE-/GRE-^ males (*n* = 14), and defeated S1PR3^GRE-/GRE-^ females (*n* = 9). Lines represent means ± SEM. *p < 0.05, **p < 0.01, ***p < 0.001; Tukey’s multiple comparisons following 3-way ANOVA. For **d**, ¥ signifies *p* < 0.05 compared to all groups except one another.

We previously demonstrated that S1PR3 mitigates defeat-induced increases in ionized calcium-binding adaptor molecule (IBA1)+ cell densities in male rats indicating increases in microglial densities [38]. Others have demonstrated that S1PR3 reduces inflammatory processes in peripheral tissue [44–46]. We found that regardless of genotype, IBA1+ cell densities are reduced in the PL and IL of females compared to males (Fig. 3a, b). In the IL of males, defeated S1PR3^GRE-/GRE-^ rats display higher IBA1+ cell densities compared to non-defeated S1PR3^GRE-/GRE-^ rats and defeated WT males (Fig. 3b, c). Therefore, GR-induced S1PR3 mitigates stress-induced increases in microglia densities in the IL of male rats. Reduced microglia densities in females compared to males might be attributed to anti-inflammatory effects of elevated corticosterone or other sex-dependent factors that do not involve S1PR3.

Monocyte concentrations [47] and neutrophil-to-lymphocyte ratios [14, 15, 22] are higher in humans with stress-related illness, although the underlying mechanisms are not well understood. S1PR3 is expressed in peripheral immune cells and reduces inflammatory processes in monocytes [45]. Monocyte concentrations are lower in WT females compared to WT males in the absence of stress. However, this effect is not observed in S1PR3^GRE-/GRE-^ males and females, indicating that this sex difference requires GR-induced S1PR3. We also found that stress reduces monocyte concentrations in WT males, but stress increases monocyte concentrations in WT females. These effects were not observed in S1PR3^GRE-/GRE-^ rats, indicating that GR-induced S1PR3 is necessary for changes in monocyte concentrations caused by stress (Fig. 3d).

We found that overall concentrations of neutrophils and lymphocytes are affected by defeat stress and sex, but not regulated by GR-induced S1PR3. Across all groups, defeat reduces neutrophil concentrations, but no genotype effects were observed (Fig. 3e). Across all groups, females displayed lower lymphocyte concentrations compared to males, but no genotype effects were observed (Fig. 3f). However, defeat increased neutrophil-to-lymphocyte ratios in S1PR3^GRE-/GRE-^, but not WT, males. This resulted in an increased neutrophil-to-lymphocyte ratio in defeated S1PR3^GRE-/GRE-^ males compared to defeated WT males (Fig. 3g). Therefore, GR-induced S1PR3 is required to prevent increases in neutrophil to lymphocyte ratios caused by stress in males. No genotype effects were observed in females. Our findings on monocytes, neutrophils, and lymphocytes indicate that GR-induced S1PR3 is necessary for changes in immune cell concentrations caused by stress.

## LC-mPFC coherence is increased in S1PR3^GRE-/GRE-^ rats and reduces sociability

**Fig. 4:**
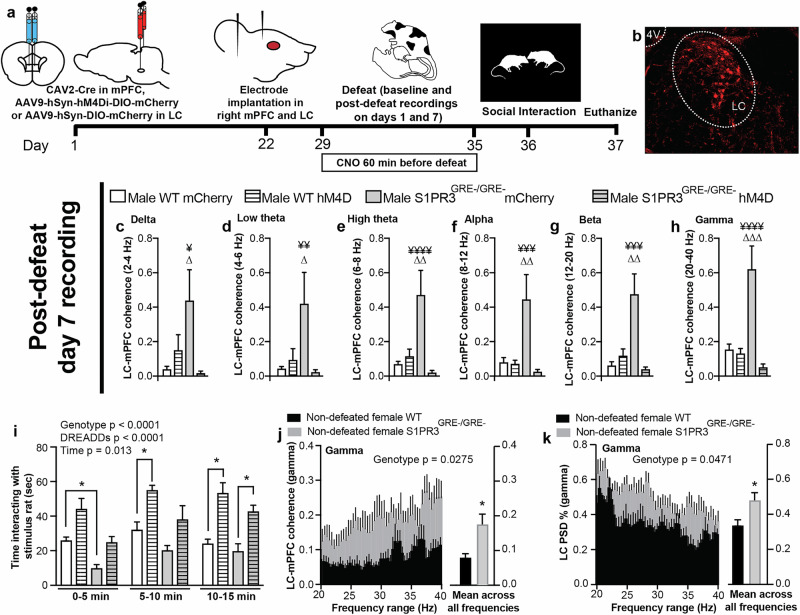
LC-mPFC coherence is increased in defeated male and non-defeated female S1PR3^GRE-/GRE-^ rats. **a** Schematic of experimental timeline. **b** mCherry expression in mPFC-projecting LC neurons in rats injected with CAV2-Cre in the mPFC and AAV9-hSyn-hM4Di-DIO-mCherry in the LC. LC-mPFC coherence in the **c** delta, **d** low theta, **e** high theta, **f** alpha, **g** beta, and **h** gamma frequency ranges. **i** Social interaction is reduced in S1PR3^GRE-/GRE-^ rats (genotype effect *p* < 0.0001) and increased in both genotypes in rats expressing hM4D (DREADDs effect *p* < 0.0001). **j** LC-mPFC coherence and **k** LC PSD% in the gamma range is increased in S1PR3^GRE-/GRE-^ females compared to WT females after seven days of handling in the absence of defeat. For **c**–**i**, WT mCherry (*n* = 7), S1PR3^GRE-/GRE-^ mCherry (*n* = 6), WT hM4D (*n* = 6), S1PR3^GRE-/GRE-^ hM4D (*n* = 5). For **j**, **k**, non-defeated WT females (*n* = 5), S1PR3^GRE-/GRE-^ females (*n* = 7). Lines represent means ± SEM. For differences between two groups, *p < 0.05. For differences compared to all other groups within a given timepoint, ¥*p* < 0.05, ¥¥*p* < 0.01, ¥¥¥*p* < 0.001; ¥¥¥¥*p* < 0.0001. For differences compared to all other timepoints for that group, ∆*p* < 0.05, ∆∆*p* < 0.01, ∆∆∆*p* < 0.001. All pre- and post-defeat recordings for Day 1 and 7 are available in Supplementary Fig. 2. For **c**–**h**, Fisher’s Least Significant Difference Test following repeated measures 3-way ANOVA for all groups and timepoints was used. For **i**, Tukey’s multiple comparison test following 3-way ANOVA. For **j**, **k**, two-way repeated measures ANOVA was used to detect genotype difference across all frequency bins.

We hypothesized that altered activation of the mPFC and/or its afferents underlies the effects of S1PR3 on social interaction in defeated males and non-defeated females. Stress increases locus coeruleus (LC) activity and increases LC-mPFC coherence in the high theta range [48]. To determine whether S1PR3 mitigates the effects of stress-activated LC afferents to the mPFC, we quantified local field potentials (LFPs) in the mPFC and LC along with their coherence, a measure of synchronous oscillations, in the delta (1.5–4 Hz), low theta (4–6 Hz), high theta (6–8 Hz), alpha (8–12 Hz), beta (12–20 Hz), and gamma (20–40 Hz) frequency ranges in WT and S1PR3^GRE-/GRE-^ males. To determine whether any changes in LFPs or coherence within specific frequency ranges were driven by mPFC-projecting LC neurons, we used Cre-dependent hM4D Designer Receptors Exclusively Activated by Designer Drugs (DREADDs) to chemogenetically inhibit mPFC-projecting LC neurons. Rats were injected with clozapine-*N*-oxide (CNO), the synthetic ligand for hM4D, 60 min prior to defeat onset every day for 7 days. 24 h later, in the absence of CNO, social interaction was assessed (Fig. 4a). In mCherry-expressing S1PR3^GRE-/GRE-^ rats across all frequency ranges, LC-mPFC coherence was increased during the day 7 post-defeat recording compared to all other groups at that timepoint and all other timepoints for that group (Fig. 4c–h, all timepoints available in Supplementary Fig. 2a–f). These findings were not observed in hM4D-expressing rats, indicating that GR-induced S1PR3 and chemogenetic inhibition of mPFC-projecting LC neurons mitigate stress-induced LC-mPFC coherence. LC-mPFC coherence is increased in the high theta range following a single defeat compared to all other timepoints in mCherry-expressing WT rats but this did not occur when LC-mPFC projections were inhibited (Supplementary Fig. 2c). During the day 1 post-defeat recording, LC-mPFC coherence in high theta and alpha is reduced in hM4D-expressing WT rats compared to mCherry-expressing WT rats (Supplementary Fig. 2c, d), confirming efficacy of the hM4D DREADDs. These results confirm previous findings that high theta LC-mPFC coherence is increased following a single defeat in WT controls [48]. Analysis of power spectral density percentages (PSD %) within the mPFC and LC did not provide any notable differences across group, time, or frequency range (Supplementary Fig. 3).

Based on increased LC-mPFC coherence in defeated S1PR3^GRE-/GRE-^ rats, we hypothesized that increased input to the mPFC from the LC during stress reduces sociability in WT and S1PR3^GRE-/GRE-^ rats. 24 h following their 7th defeat, social interactions were assessed in mCherry- and hM4D-expressing WT and S1PR3^GRE-/GRE-^ rats. Genotype (*p* < 0.0001), DREADDs (*p* < 0.001), and Time (*p* = 0.013) effects are observed as social interaction is higher in hM4D-expressing rats regardless of genotype and social interaction was lower in S1PR3^GRE-/GRE-^ rats regardless of whether mPFC-projecting LC neurons are inhibited or not (Fig. 4i). This confirms reduced social interaction in S1PR3^GRE-/GRE-^ rats and demonstrates that chemogenetic inhibition of mPFC-projecting LC neurons during stress increases subsequent sociability in the absence of any manipulation to ongoing neuronal activity.

**Fig. 5:**
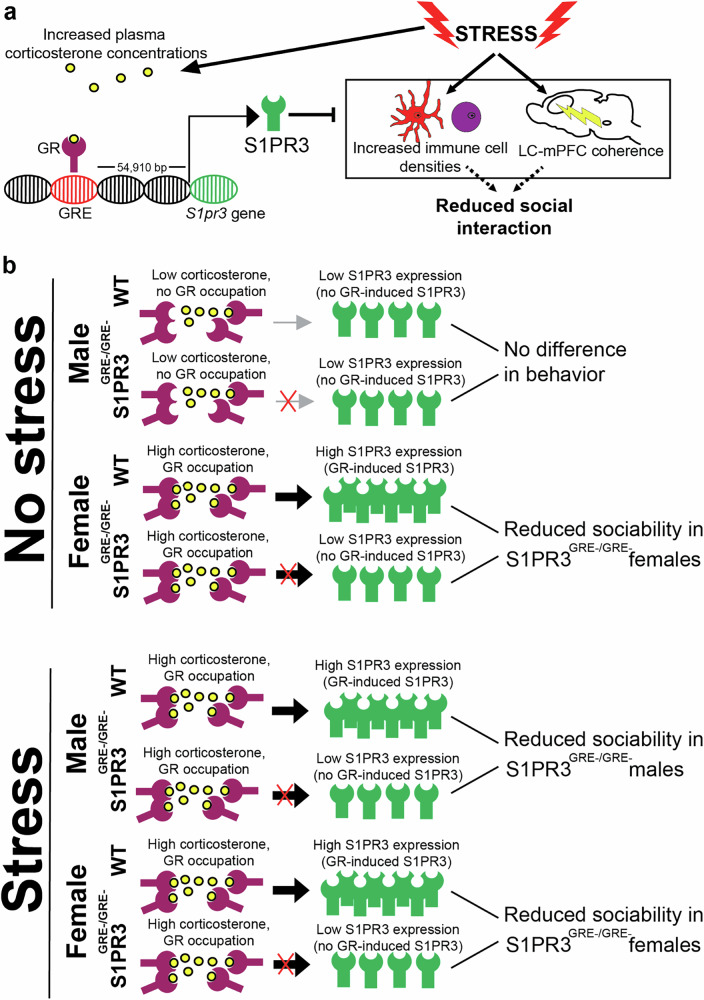
Glucocorticoid receptors increase S1PR3 to prevent reductions in sociability caused by stress. **a** Graphical summary of results showing that corticosterone increases S1PR3, which mitigates increases in immune cell densities and LC-mPFC coherence normally caused by stress thereby preventing stress induced reductions in sociability. **b** Sociability in non-stressed WT and S1PR3^GRE-/GRE-^ males is similar because S1PR3 levels are similar. Compared to non-stressed males, circulating corticosterone levels are higher in stressed males, non-stressed females, and stressed females. This activates GRs, which increase S1PR3 transcription in WT, but not S1PR3^GRE-/GRE-^ rats. The relatively lower levels of S1PR3 causes reduced sociability in S1PR3^GRE-/GRE-^ rats compared to their respective WT controls.

We hypothesized that LC-mPFC coherence is increased in S1PR3^GRE-/GRE-^ females under baseline conditions because S1PR3^GRE-/GRE-^ females display reduced sociability in the absence of stress. LC-mPFC coherence is increased in the gamma range of S1PR3^GRE-/GRE-^ females compared to WT female littermates (Fig. 4j). This may be driven by increased LC PSD % in the gamma range, which is increased in S1PR3^GRE-/GRE-^ females compared to their female WT littermates (Fig. 4k). No other notable changes in other frequency ranges were observed in females (Supplementary Fig. 4). Together, these findings demonstrate that LC-mPFC coherence is increased in defeated S1PR3^GRE-/GRE-^ males and non-defeated S1PR3^GRE-/GRE-^ females. As a whole, our findings indicate that GR-induced S1PR3 is required to mitigate stress-induced inflammatory processes and maintain sociability by preventing LC-mPFC coherence in stressed male rats and females under baseline conditions (Fig. 5a). Compared to non-stressed males, circulating corticosterone levels are higher in stressed males, non-stressed females, and stressed females. This activates GRs, which increase S1PR3 transcription in WT, but not S1PR3^GRE-/GRE-^ rats. The relatively lower levels of S1PR3 causes reduced sociability in S1PR3^GRE-/GRE-^ rats compared to their respective WT controls (Fig. 5b). Our findings support the hypothesis that circulating plasma corticosterone concentrations, which are elevated in females and stressed males compared to non-stressed males, increase the expression of S1PR3. The predicted GR binding site near the S1PR3 gene that is deleted in S1PR3^GRE-/GRE-^ rats is important for increasing S1PR3 expression, reducing inflammatory processes, maintaining low LC-mPFC coherence, and promoting sociability. Because baseline corticosterone concentrations are higher in females compared to males, preventing GR-induced S1PR3 causes a more severe phenotype in S1PR3^GRE-/GRE-^ females compared to their WT controls. Stress is required to observe differences in behavior between WT and S1PR3^GRE-/GRE-^ males because GRs are not increasing S1PR3 in the absence of stress.

## Discussion

Here we show that GR-induction of S1PR3 mitigates stress-induced inflammatory processes in the mPFC, mitigates stress induced increases in coherence of activity between the LC and mPFC, and mitigates reductions in sociability in stressed males. These effects were also observed in females but under both baseline and stress conditions. We hypothesize that effects were observed in females in the absence of stress because baseline corticosterone levels in females are 5–10 fold higher than those of males [42, 49, 50] and within the range of stressed males [39]. This causes S1PR3 expression to be higher in the mPFC and blood of WT females compared to WT males in the absence of stress. This sex difference was not observed in S1PR3^GRE-/GRE-^ rats, supporting the hypothesis that corticosterone-activated GRs increase S1PR3 in females under baseline conditions. We attribute reduced S1PR3 levels in S1PR3^GRE-/GRE-^ rats to impaired transcription mediated by GRs because (1) the sequence we targeted for deletion was originally identified using a genome-wide analysis of GR binding sites in mice [41], (2) we previously demonstrated that GR knockdown in the mPFC reduces S1PR3 in defeated WT male rats [38], and (3) we show here that adrenalectomy lowers S1PR3 in the IL of non-defeated WT females. We also showed that LC-mPFC coherence is increased across all frequency ranges following the 7th defeat in S1PR3^GRE-/GRE-^ males and increased in the gamma frequency range during a baseline recording in S1PR3^GRE-/GRE-^ females. Inhibition of mPFC-projecting LC neurons during daily stress reduces stress-induced coherence between the LC and mPFC and increases subsequent sociability in WT and S1PR3^GRE-/GRE-^ males.

Importantly, reduced social interaction in S1PR3^GRE-/GRE-^ females compared to WT females was even observed under baseline conditions, indicating a more severe phenotype in S1PR3^GRE-/GRE-^ females compared to males. We previously demonstrated that stressinduced S1PR3 mitigates the development of maladaptive behavior in males. We confirmed this finding here and found that GR-induced S1PR3 may play a more important role in females as it regulates S1PR3-mediated processes, including social behavior, inflammation, and LC-mPFC coherence, even under baseline conditions.

Inflammatory processes induced by stress in the mPFC might be driven by activation of the LC and sympathetic nervous system as norepinephrine increases microglial reactivity [32] and cytokine production [51] in the brain and increases NFkB-mediated transcription in peripheral immune cells [31]. Consistent with previous findings in S1PR3 knock-down rats [38], we report that densities of IBA1+ microglia are increased in the IL of defeated S1PR3^GRE-/GRE-^ males compared to defeated WT males. Monocyte concentrations are higher in defeated male and non-defeated female S1PR3^GRE-/GRE-^ rats compared to their WT counterparts, indicating that GR-induced S1PR3 mitigates stress-induced increases in monocytes in males and maintains low monocyte concentrations in females under baseline conditions. We were interested in the neutrophil-to-lymphocyte ratio for each rat because it is a biomarker for PTSD [22] and MDD [15, 23], especially in males [14]. The neutrophil-to-lymphocyte ratio is thought to be a measure of acute inflammatory processes compared to baseline immune function as neutrophils have a half-life of around 6–12 h [52, 53] whereas the lymphocyte half-life is on the order of weeks [54–56]. We found that the neutrophil-to-lymphocyte ratio was increased in defeated male S1PR3^GRE-/GRE-^ rats compared to non-defeated S1PR3^GRE-/GRE-^ males and defeated WT males. This suggests that GR-induced S1PR3 mitigates stress-induced neutrophil-to-lymphocyte increases in males.

Coherence is a measure of local field potential synchrony between two brain regions and is generally interpreted as more effective communication between those brain regions, although activity driven by a common input from a third brain region can also underlie coherence. The LC is the primary source of norepinephrine in the brain [57], it is activated by social defeat [48], and it is an important mediator of mPFC function [58, 59]. We confirmed previous findings that a single defeat increases coherence in the high theta range following a single defeat in WT controls [48]. This increase does not occur when mPFC-projecting LC neurons are chemogenetically inhibited. Therefore, defeat-induced LC-mPFC coherence, at least in the high theta range, can be primarily attributed to mPFC-projecting LC neurons rather than common afferents. LC-mPFC coherence was increased during the day 7 post-defeat recording in mCherry-, but not hM4D-, expressing S1PR3^GRE-/GRE-^ rats across all frequency ranges. Here, effects were not observed on day 1, presumably because S1PR3 expression is similar in WT and S1PR3^GRE-/GRE-^ males under baseline conditions. Effects might only be observed during the post-defeat recording because LC neurons are activated by stress [48, 60], causing prolonged activation of the mPFC [61, 62]. This finding indicates that normally, GR-induced S1PR3 mitigates stress-induced LC-mPFC coherence.

We found that chemogenetically inhibiting mPFC-projecting LC neurons only during social defeat, without administering CNO during social interaction testing, increases subsequent social interaction in WT and S1PR3^GRE-/GRE-^ rats in the absence of any direct manipulations to these neurons during social interaction testing. Chemogenetically inhibiting these neurons during social interaction could have affected other processes, like attention [59], making it difficult to parse out effects caused by stress. The precise mechanism(s) by which stress-activated mPFC-projecting LC neurons reduce sociability are not known. One potential mechanism is increased inflammatory processes as TNFα is increased in the mPFC of stressed rodents [38, 63], especially when S1PR3 is knocked down [38]. TNFα is important for social interaction as pharmacological inhibition of TNFα ameliorates sociability [38, 64]. Norepinephrine may be an important factor driving stress-induced cytokine production in the mPFC as it increases microglial reactivity and cytokine production in the brain [32, 51]. Therefore, increased cytokine expression may represent one important factor caused by stress-induced norepinephrine that promotes social anxiety behaviors.

We wanted to better understand the mechanisms by which S1PR3^GRE-/GRE-^ females display reduced sociability under baseline conditions compared to female WT controls. Because LC-mPFC coherence is increased in defeated S1PR3^GRE-/GRE-^ males and important for sociability, we hypothesized that LC-mPFC coherence might be increased in S1PR3^GRE-/GRE-^ females in the absence of stress. We found that LC-mPFC coherence was increased in the gamma range in S1PR3^GRE-/GRE-^ females compared to their WT controls. This might be driven by LC activity in the gamma range as S1PR3^GRE-/GRE-^ females displayed higher gamma power in the LC compared to their WT counterparts. This increase in LC activity in the absence of stress in S1PR3^GRE-/GRE-^ females, which is not observed in S1PR3^GRE-/GRE-^ males, might be related to increased dendritic arborization in the LC of females compared to males [65].

It is well established that GRs underlie certain adverse effects of stress, like promoting the generalization of fear memories [66], reducing hippocampal volume [67], or increasing hypertrophy of amygdala dendrites [68]. However, GRs also have properties that mitigate maladaptive changes in behavior that are normally caused by stress. In rodents, GR knockout from forebrain neurons causes depression-like behavior and heightened activation of the neuroendocrine stress response [69], which is likely attributed to impaired negative feedback of the HPA axis [69, 70]. GRs also exert strong anti-inflammatory effects [29, 71], making them important for mitigating stress-induced inflammatory processes. GRs control multiple mechanisms that reduce pro-inflammatory transcription [72–74]. Here, we show that one of the genomic sites required for the anti-inflammatory effects of GRs is proximal to the S1PR3 gene. We also show that GR-induced increases in S1PR3 are important for mitigating LC-mPFC coherence. Reducing LC-mPFC coherence promotes sociability as chemogenetically inhibiting mPFC-projecting LC neurons during stress increases subsequent social interaction. Therefore, while some of the mechanisms regulated by GRs contribute to maladaptive changes in behavior, other mechanisms regulated by GRs in other cell types or brain regions can mitigate maladaptive changes that would otherwise be caused by stress.

Future work will focus on the precise epigenetic mechanisms by which GRs regulate S1PR3 expression. Because the GRE deleted in S1PR3^GRE-/GRE-^ rats is more than 50,000 bp upstream of the S1PR3 gene, distal interactions involving multiple proteins are likely required for GR-induced S1PR3 expression [75]. Among other histone modifications [29, 72], GRs recruit histone acetyl transferases to increase gene expression [76, 77]. Other epigenetic mechanisms that increase gene expression, like histone methylation, may also be involved. An important caveat is that deletion of the GRE upstream of the S1PR3 gene may also modulate the expression of genes other than S1PR3. Further, although we targeted a predicted GRE based on previous work that used ChIP-seq [41] and we previously found that GRs are required for stress-induced S1PR3 [38], it is difficult to know whether other factors bind to the region deleted in S1PR3^GRE-/GRE-^ rats that might also be important for increasing S1PR3 expression. Other factors might be involved in increasing S1PR3 in non-stressed WT females compared to males. While baseline concentrations of total plasma corticosterone are 5–10-fold higher in female rats compared to males [42, 43], females also express 2-3-fold higher levels of corticosteroid binding globulin (CBG) [78]. CBG lowers, but does not eliminate, the concentration of free corticosterone available to bind GRs [79–81]. Because free corticosterone levels in the brain are subject to circadian and ultradian rhythms [82], it is possible that periodic increases in corticosterone increase S1PR3 expression, even if free corticosterone concentrations in the brain are less pronounced than total plasma corticosterone concentrations. Supporting this, adrenalectomy in wild-type females reduces S1PR3 protein in the mPFC within seven days. Future experiments will investigate the role of corticosterone dynamics and CBG on S1PR3 in males and females.

In sum, three main novel findings were observed. First, we show that a specific GRE is important for regulating anti-inflammatory processes, neuronal activity, and behavior. It is well established that GRs bind to GREs throughout the genome and regulate gene expression [41, 72, 77], reduce inflammatory processes [29, 37, 83], and modulate brain function and behavior [84–86], but determining a causative role for GREs can only be accomplished by deleting or mutating GREs in vivo. A GRE deletion has been used in mice to investigate effects on circadian rhythms and glucose homeostasis [87]. However, to the best of our knowledge, a single GRE had not been implicated in systemic inflammatory processes, coherence between brain regions, or stress-related behavior. CRISPR/Cas9 technology has made this more accessible, which is particularly beneficial in rats since few genetic rat models exist due to the difficulty to altering the rat genome. Second, S1PR3^GRE-/GRE-^ females display reduced social interaction compared to WT controls even in the absence of stress. This indicates that S1PR3 is an important neural substrate promoting sociability in females under baseline conditions. Third, we demonstrate that GR-induced S1PR3 mitigates increased LC-mPFC coherence caused by stress. This might have important implications for attention and cognitive flexibility, processes that are dependent on the mPFC and LC [58, 88]. Further, we show that LC afferents to mPFC contribute to reductions in social interactions caused by stress. Together, these findings identify a novel genomic locus that mitigates inflammatory processes and promotes stress resilience by mitigating stress-induced coherence in a circuit that contributes to social anxiety-like behavior.

## Methods

### Generation of S1PR3^GRE-/GRE-^ rats

S1PR3^GRE-/GRE-^ rats were generated under the consultation of the University of Pennsylvania CRISPR/Cas9 Targeting Core. Chromatin immunoprecipitation sequencing had previously identified a GR binding site 34,519 base pairs upstream of the *S1pr3* gene in mice. Notably, 16% of GR binding sites are greater than 50 kb from the transcriptional start or stop sites, with only 5% being within 5kb [41]. Using the BLAST-Like Alignment Tool, we identified the homologous sequence in rats. For the generation of S1PR3^GRE-/GRE-^ rats, a 1445 base pair sequence on chromosome 17 (5′-13738152–13739596-3′), was deleted. The 3′ end of this sequence is 54,910 base pairs upstream of the first base pair on the 5′ end of the DNA encoding the first exon of the S1PR3 transcript. Guide (g)RNA and Cas9 were microinjected into a fertilized embryo. The 5′ gRNA target sequence was GTGCCCTCTGTAGAACTGCGGGG and the 3′ gRNA target sequence was GAATTGGTCTGTGACGTAGGAGG. Based on genome-wide ChIP-seq that identified the GRE near the S1PR3 gene [41], the putative GRE is ACAGTATGGCAGCTCTGTAGATGACCC, which begins 689 base pairs downstream of the end of the 5′ gRNA. The overlapping junction following deletion is AGTGTGCCCTCTGTAGAACTAGGAGGAGCC. Primers used to confirm knockout were CAAGATTCCCAAGAGGATGC (forward) and CCTCAATACAGGGCTCTTGC (reverse). Four heterozygous founders were created and paired for mating to create homozygous pups. Genotyping of ear punches for all pups was performed by Transnetyx. No experiments were performed on any rats until the F_4_ generations in an effort to avoid any off-target effects.

### Animals

Adult male and female S1PR3^GRE-/GRE-^ rats and WT littermate were used in all experiments. No heterozygotes were used in these studies. Rats were group housed until they were either defeated, served as a novel cage non-defeated control, or underwent surgery. Rats were defeated or served as novel cage controls at 12–14 weeks of age. For experiments requiring virus-mediated gene transfer and in vivo electrophysiology, surgery was performed on 8-week-old rats. Long-Evans retired breeder males were purchased from Charles River Laboratories (Wilmington, MA, USA; 650–850 g) served as residents for male defeat. Lactating females were purchased from Charles River Laboratories and served as residents for female defeat. Residents were singly housed upon arrival. Females arrive on gestational day 18 and are singly housed. For females, defeats begin when the resident has pups that are three days old. Pups are removed just before defeat and returned just after defeat. Female intruders are removed after no more than five attacks because female residents remain aggressive even after intruder submission. Once experimental procedures began, S1PR3^GRE-/GRE-^ rats and their WT littermates were singly housed in polycarbonate cages with standard bedding and with food and water available *ad libitum*. Animals were kept on a 12-h light–dark cycle with lights on at 06:15 and lights off at 18:15 in a temperature-controlled vivarium for at least 5 days prior to administration of any stress protocols. All experiments took place during the inactive phase between 1000 and 1400 h. Rats were euthanized by rapid decapitation and their brains were immediately snap-frozen in 2-methylbutane. Each day following social defeat, the rats were inspected by the experimenter and an animal technician. Any signs of pain (blood, limping, etc.) were assessed by a veterinarian who recommended euthanasia if symptoms were too severe. All data represent combined results replicated from one or two cohorts. Experiments were performed in compliance with all relevant ethical regulations for animal testing and research. Experiment protocols followed the NIH Guide for the Care and Use of Laboratory Animals and were approved by the Children’s Hospital of Philadelphia Research Institute’s Animal Care and Use Committee.

### Chromatin immunoprecipitation (ChIP)

ChIP was performed similarly to described methods [42, 89–91] according to the ChIP kit (EMD Millipore, 17–295) manufacturer’s instructions. Prefrontal cortices were subdissected and fixed in 1% formaldehyde. Samples were sonicated to generate genomic fragments 200–1000 bp in length and pre-cleared with Protein A beads prior to incubation with a rabbit-anti-GR monoclonal primary antibody (Cell Signaling, D6H2L, 12041S) 4 °C overnight. The antibody-chromatin complex was immunoprecipitated with Protein A beads, washed with a series of buffers (Millipore), and then chromatin was eluted and reverse cross-linking performed with Proteinase K. DNA was purified via phenol-chloroform extraction. Final DNA concentration was measured using the NanoDrop 2000. qPCR was performed with an ABI 7500 PCR machine using SYBR green as a fluorophore. Primers used to amplify the GRE near the *S1pr3* gene were CTGGAACTGTACCCAACGCT (forward) and AAACTAGCCGAGAAGCCAGG (reverse). The GAPDH primers GACATGCCGCCTGGAGAAAC (forward) and AAGCAGTTGTCCTGTTGGGA (reverse) were used for the housekeeping control. S1PR3 GRE amplification was not observed in S1PR3^GRE-/GRE-^ rats, presumably because this sequence was deleted.

### Resident-intruder model of social defeat

The resident-intruder model of social defeat paradigm was performed as previously described [38, 39, 48]. Rats were randomly assigned to either a social defeat or novel cage control group for 7 consecutive days. During each episode of social stress, a rat was placed into the home cage territory of an unfamiliar Long-Evans resident previously screened for high aggression. A typical agonistic encounter resulted in intruder subordination or defeat, signaled by the intruder assuming a supine position for 3 s. After defeat, a wire mesh partition was placed in the cage to prevent physical contact between the resident and intruder but allowing visual, auditory, and olfactory contact for the remainder of the 30 min defeat session. Latency to assume a submissive posture (defeat) was recorded and averaged over the seven daily defeat exposures. Rats that were not attacked were not included in defeat latency analysis for that day. If an intruder resisted defeat for 15 min, the resident and intruder were separated with the wire partition for the remainder of the session. Controls were placed behind a wire partition in a novel cage for 30 min daily. Rats were returned to their home cage after each session.

### Immunohistochemistry

Brains were sectioned on a cryostat at 20 µm. The following primary antibodies were used: rabbit anti-S1PR3/Edg3 (bs-7541R, 1:100, BIOSS), rabbit anti-IBA1 (019-19741, Wako, 1:250), and rabbit anti-mCherry (ab167453, 1:200, Abcam). The following secondary antibodies were used: donkey anti-rabbit (Alexa Fluor ® 488, Abcam, ab150073), donkey anti-rabbit (Alexa Fluor ® 594, Abcam, ab150076), and biotinylated donkey anti-rabbit (Jackson Laboratories, 711-065-152). All secondary antibodies were used at a concentration of 1:200. All immunohistochemical comparisons of protein expression were from assays performed at the same time with the same working solutions. For staining with 3,3′ -diaminobenzidine (DAB), further amplification was accomplished using Avidin-Biotin Complex (Vectastain). DAB (Sigma) was used as a chromagen. For quantifying S1PR3, two sections between Bregma + 3.0 and Bregma + 3.4 mm were chosen for analysis. Here, optical density in layer 1 adjacent to the midline was used as a background to be subtracted from neuronal fluorescence. An 8 × 8 grid was superimposed on each image. S1PR3 immunoreactivity (IR) was quantified in three neurons closest to the grid lines in dorsal, central, and ventral mPFC layers 2/3, 4/5, and 6 (9 cells/section). Means of these cells were created for each section. Sections in which the tissue was not wholly intact or damaged were discarded from analysis.

### Stereotaxic virus injections

Cre-dependent AAV9-hSyn-DIO-hM4D-HA-mCherry or control AAV9-hSyn-DIO-HA-mCherry were purchased from the University of North Carolina Vector Core. CAV2-Cre was purchased from Institut de Génétique Moléculaire de Montpellier, University of Montpellier. Rats were weighed and anesthetized with a ketamine/acepromazine/xylazine cocktail (1/0.2/0.02, 1 mL/kg). The LC (500 nL, A/P: Lamda −3.7, D/V: 5.1 mm ventral, M/L: ± 1.4 mm) and mPFC (1 µL, A/P: Bregma + 3.2 mm, D/V: 4.4 mm, M/L: ± 0.5 mm) were injected over the course of ten minutes. Surgeries were performed 19–21 days prior to restraint to allow for overexpression. For animal inclusion, mCherry was required to be detected in the LC without significant (>10 cells) expression in other regions in the pons or in the cerebellum. One rat was excluded from the hM4D-expressing S1PR3^GRE-/GRE-^ group for this reason. Of note, seven S1PR3^GRE-/GRE-^ rats but only one WT control died during surgery due to excessive blood loss. This may be due to a clotting impairment exhibited by S1PR3^GRE-/GRE-^ rats.

### Social interaction

Animals were placed in an open field black box (70 cm × 70 cm) with a sex- and age-matched stimulus rat of the same strain and of a similar size and allowed to interact for 15 min. Time interacting with the stimulus rat was defined by the time the rat was actively investigating the stimulus rat with its snout closer than 3 cm away (approximately the length of the snout of the rat) from the stimulus rat. Each interaction was videotaped and coded for social interaction time by 2 coders who were blind to the experimental conditions.

### Local field potential recordings

21 days following virus surgery, the right mPFC (A/P: Bregma + 3.2 mm, D/V: 4.4 mm, M/L: 0.5 mm left) and LC (A/P: Lamda −3.7, D/V: 5.1 mm ventral, M/L:1.4 mm right) had custom recording electrodes (MicroProbes, Part# PI(7 mm)0030.1B10) placed within 0.5 mm of the virus injection sites. Cables connected the head stage to the data acquisition system. Rats recovered for 6 days and were subjected to a 10-minute acclimation recording in their home cage. Beginning 24 h later, rats were injected with clozapine-*N*-oxide (CNO, 2 mg/kg, intraperitoneal) 60 min prior to defeat for 7 consecutive days. 10 min baseline and 10 min post-defeat recordings were performed on the first and seventh days of defeat. Cables connected the head stage to the data acquisition system. Pre-defeat baseline recordings were done in the intruder’s home cage. The cables were then disconnected and the rat was placed in the resident’s cage for defeat. Post-defeat recordings occurred after the physical interaction, while the intruder was in resident’s cage but was physically separated from the resident by the wire partition, which maintained visual, olfactory, and auditory communication between resident and intruder. Electrode recordings in the mPFC were amplified at a gain of 5000 Hz, bandwidth of 1–150 Hz. Raw mPFC and LC traces were time stamped in Spike2 to remove noise and converted to Power Spectra Density (PSD) plots indicating the relative power and coherence in 128 frequency bins from 0–50 Hz using Neuroexplorer (Nex Technologies, Madison, AL).

### Blood collection and isolation of mRNA from whole blood

400 µL of tail blood was collected in RNAprotect Animal Blood Tubes (Qiagen, cat. no. 76554) 24 h after a 7th day of social defeat. Blood mRNA was isolated using the RNeasy Protect Animal Blood Kit (Qiagen, cat. no. 73224) according to the manufacturer’s instructions.

### qRT-PCR

Reverse transcription was performed using a High-Capacity cDNA Reverse Transcription kit (4368814, Thermo Fischer Scientific). qPCR was performed with an ABI 7500 PCR machine using SYBR Green as a fluorophore. Primers used to amplify cDNA were rat *Gapdh* (forward: 5′-AGACAGCCGCATCTTCTTGT- 3′, reverse: 5′- CTTGCCGTGGGTAGAGTCAT-3′) and rat *S1PR3* (forward: 5′-CCTCATCACCACCATCCTCT- 3′, reverse: 5′-CCCTGAGGAACCACACTGTT-3′) as in our previous work [38].

### Hematology

90 µL of trunk blood was added to a tube containing 10 µL of ethylenediaminetetraacetic (EDTA). Hematology was performed by the Children’s Hospital of Philadelphia Translational Research Core to quantify concentrations of each blood cell type based on side and forward scatter. Flow cytometry was performed on a Sysmex XT automated hematology analyzer.

### Statistical analyses

Statistical analyses were performed using Prism 8. Raw means are presented in Supplementary Table 1, statistical analyses and significant post-hoc differences are presented in Supplementary Table 2. Group sizes were determined by referencing group sizes from our previous experiments, which measured similar endpoints in rats following S1PR3 knockdown [38]. Investigators were blinded to the group each sample belonged to for each endpoint. Differences between means were assessed using a two-tailed, unpaired Student’s t test unless otherwise indicated. Two-way ANOVA with p-corrected Tukey’s multiple comparisons post-hoc testing was used to assess difference among groups in analyses with two factors. For analyzing immune, immunohistochemistry, and behavioral data with three factors, three-way ANOVA with p-corrected Tukey’s multiple comparisons post-hoc testing was used to assess group differences. Post-hoc differences in PSDs and coherence were analyzed using Fisher’s Least Significant Difference testing following three-way ANOVA. Data beyond three standard deviations from the mean were considered outliers and discarded from analysis. One rat was removed due to abnormal immune cell counts.

## Supplementary information

Supplemental Figure 1

Supplemental Figure 2

Supplemental Figure 3

Supplemental Figure 4

Supplemental Table 1

Supplemental Table 2

## Data Availability

Individual data points are graphed in each main and supplemental figure. Source data files that support the findings of this study are available from the corresponding author upon request.
