# Supplementary material for: A single glucocorticoid response element regulates sociability in a sex-specific manner
Source: Mol Psychiatry. 2025 Aug 25;31(2):714–25. doi: 10.1038/s41380-025-03158-y (PMC12815654; doi:10.1038/s41380-025-03158-y)
Supplement: Supplementary file 1 — Supplemental Figure 1 [file 41380_2025_3158_MOESM1_ESM.docx]

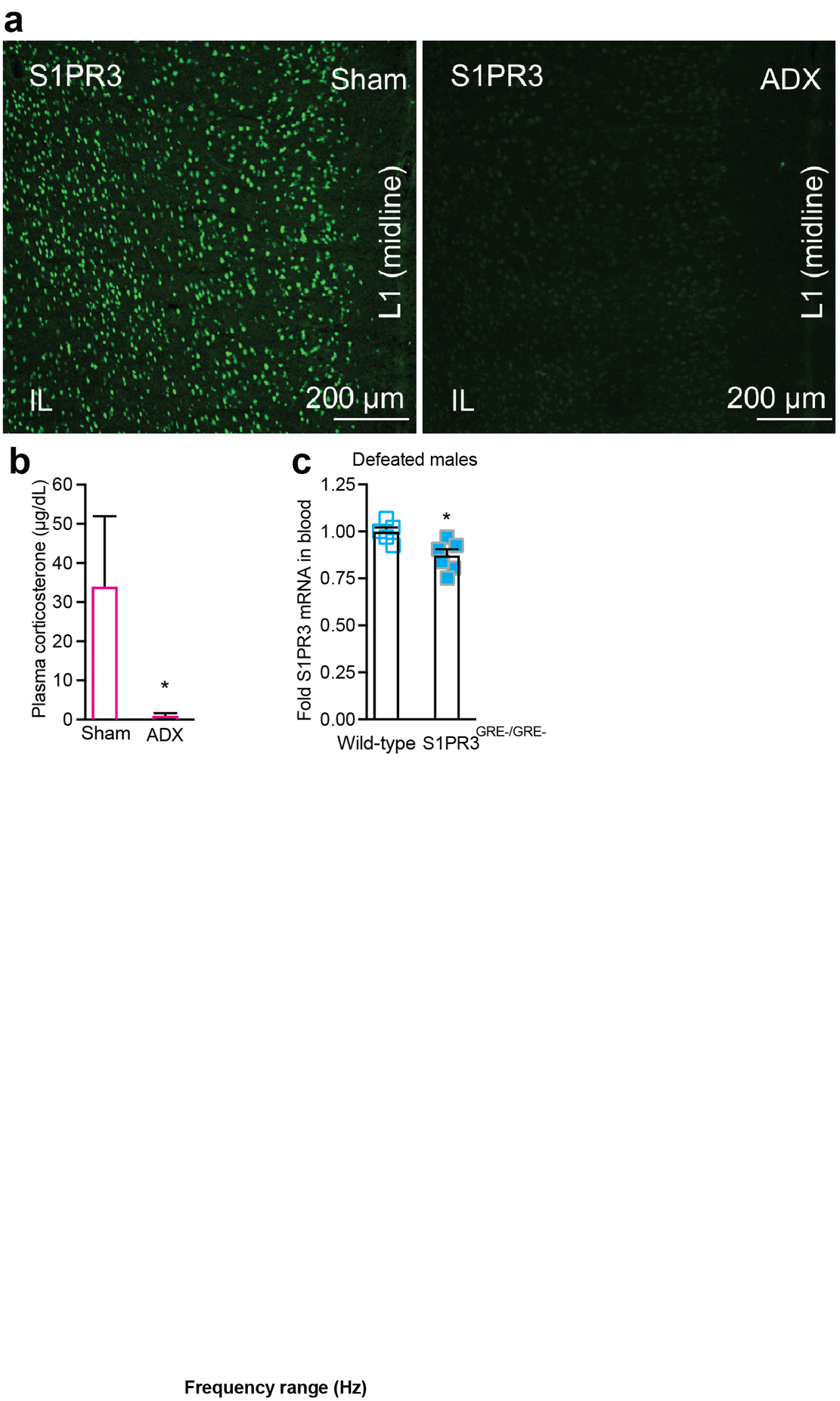


**Supplementary Figure 1. Adrenalectomy in females reduces plasma corticosterone concentrations.** (**a**) Images of S1PR3 in the IL of sham control and adrenalectomized (ADX) non-defeated females. (**b**) Plasma corticosterone concentrations in sham control (n=3) and bilaterally adrenalectomized (n=6, ADX) non-defeated females. (**c**) S1PR3 mRNA in whole blood collected from defeated WT males (n=5) and defeated S1PR3^GRE-/GRE-^ males (n=6). Bars represent means ± SEM. *p<0.05; unpaired two-tailed Student’s t-test.
