# Supplementary material for: A single glucocorticoid response element regulates sociability in a sex-specific manner
Source: Mol Psychiatry. 2025 Aug 25;31(2):714–25. doi: 10.1038/s41380-025-03158-y (PMC12815654; doi:10.1038/s41380-025-03158-y)
Supplement: Supplementary file 2 — Supplemental Figure 2 [file 41380_2025_3158_MOESM2_ESM.docx]

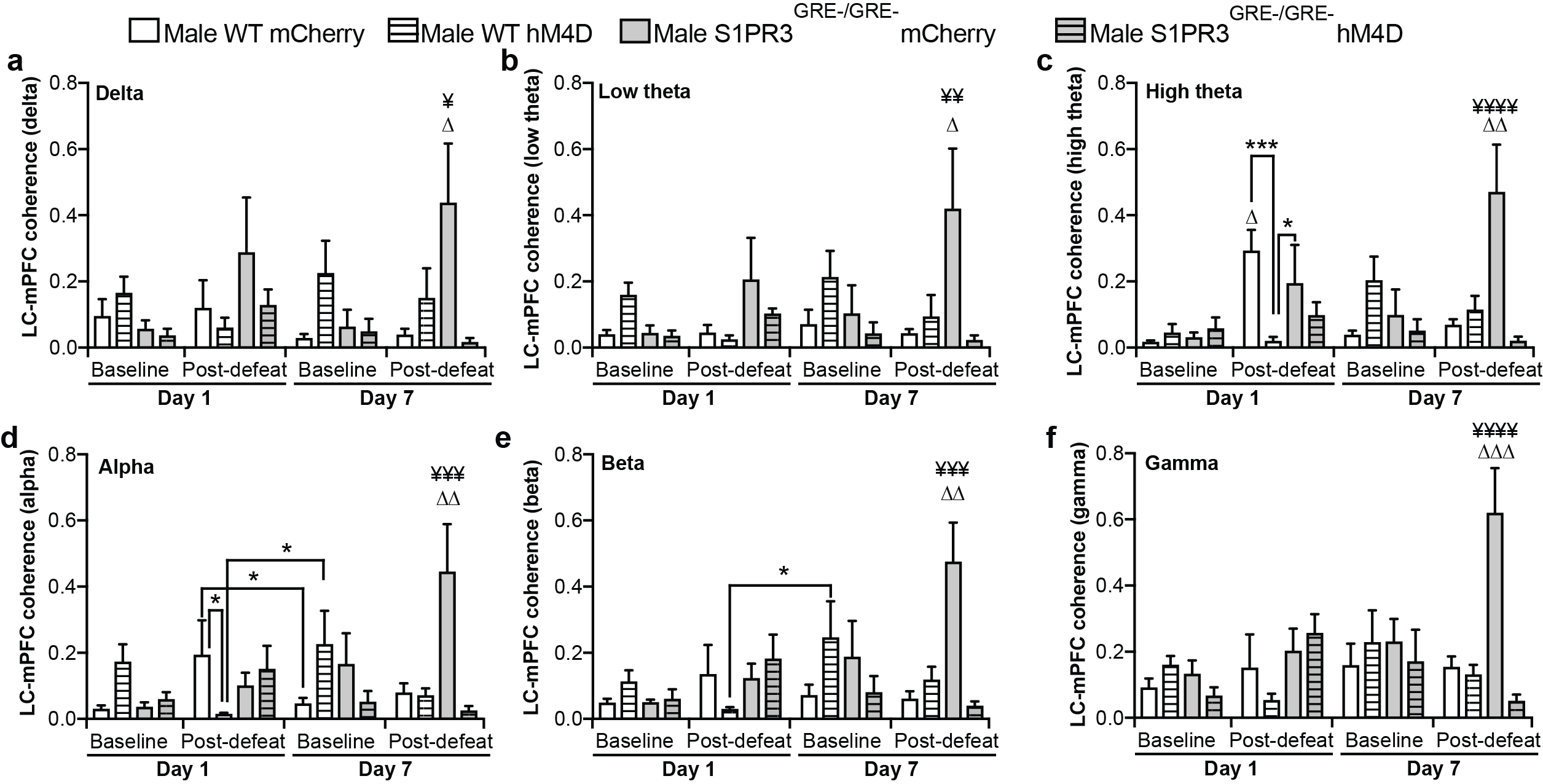


**Supplementary Figure 2. LC-mPFC coherence in defeated mCherry and hM4D wild-type and S1PR3^GRE-/GRE-^ males.** LC-mPFC coherence in the (**a**) delta, (**b**) low theta, (**c**) high theta, (**d**) alpha, (**e**) beta, and (**f**) gamma frequency ranges. WT mCherry (n=7), S1PR3^GRE-/GRE-^ mCherry (n=6), WT hM4D (n=6), S1PR3^GRE-/GRE-^ hM4D (n=5). For differences between two groups, *p<0.05, **p<0.01, ***p<0.001. For differences compared to all other groups within a given timepoint, ¥p<0.05, ¥¥p<0.01, ¥¥¥p<0.001; [¥¥¥¥p<0.0001](file:////p%3c0.0001). For differences compared to all other timepoints for that group, ∆p<0.05, ∆∆p<0.01, ∆∆∆p<0.001. Fisher’s Least Significant Difference Test following repeated measures 3-way ANOVA was used.
