# Supplementary material for: A single glucocorticoid response element regulates sociability in a sex-specific manner
Source: Mol Psychiatry. 2025 Aug 25;31(2):714–25. doi: 10.1038/s41380-025-03158-y (PMC12815654; doi:10.1038/s41380-025-03158-y)
Supplement: Supplementary file 3 — Supplemental Figure 3 [file 41380_2025_3158_MOESM3_ESM.docx]

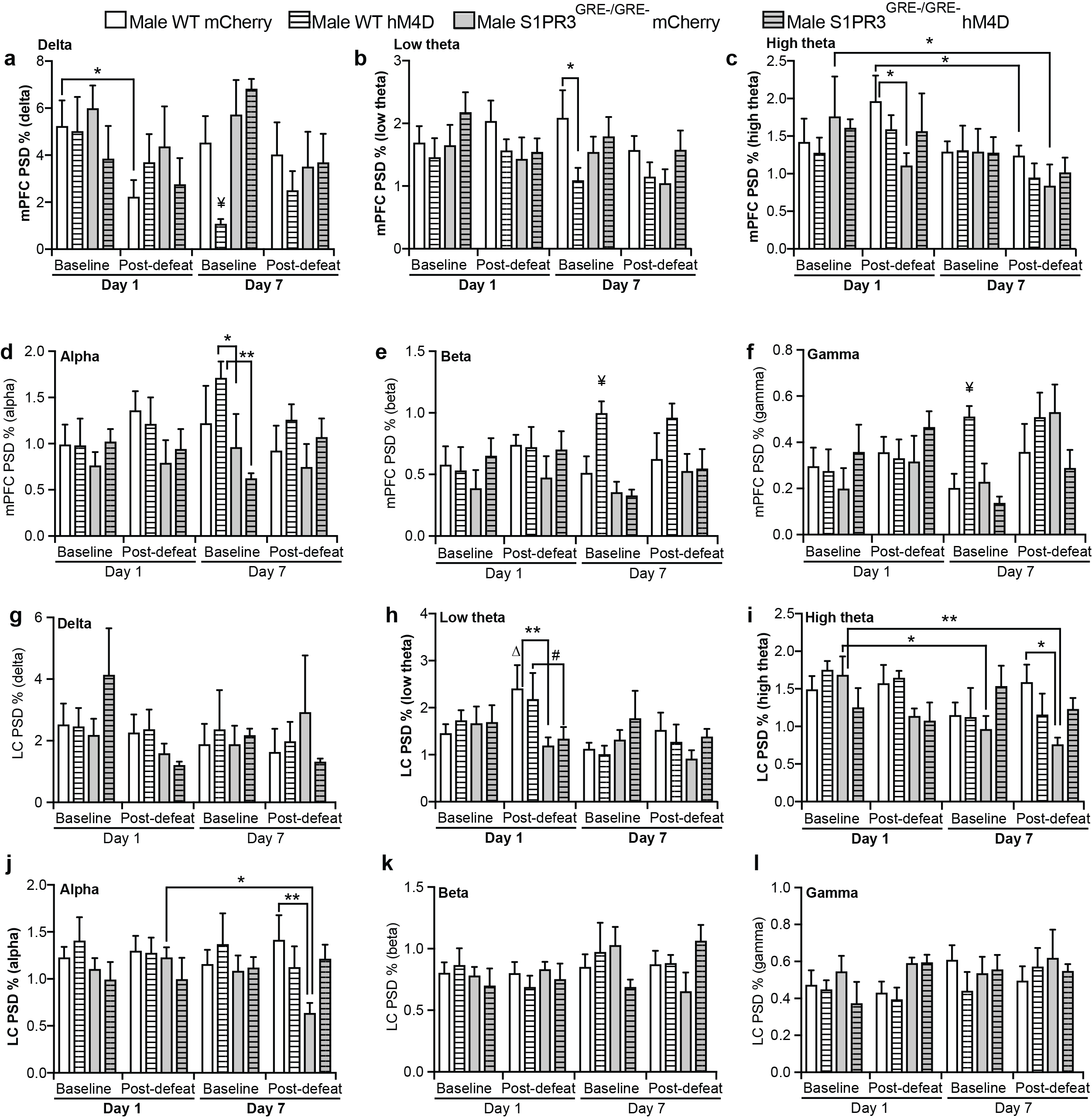


**Supplementary Figure 3. Power spectral density percentages in the mPFC and LC of defeated male wild-type and S1PR3^GRE-/GRE-^ rats.** Power spectral density percentages in the mPFC in the (**a**) delta, (**b**) low theta, (**c**) high theta, (**d**) alpha, (**e**) beta, and (**f**) gamma frequency ranges. Power spectral density percentages in the LC in the (**g**) delta, (**h**) low theta, (**i**) high theta, (**j**) alpha, (**k**) beta, and (**l**) gamma frequency ranges. For all panels and timepoints, WT mCherry (n=7), S1PR3^GRE-/GRE-^ mCherry (n=6), WT hM4D (n=6), S1PR3^GRE-/GRE-^ hM4D (n=5). Lines represent means ± SEM. For post-hoc group and timepoint differences, #p<0.010, *p<0.05, **p<0.01; ¥p<0.05 compared to all groups at that timepoint; ∆p<0.05 compared to all other timepoints for that group as assessed by Fisher’s Least Significant Difference Test following 3-way ANOVA.
