# Supplementary material for: A single glucocorticoid response element regulates sociability in a sex-specific manner
Source: Mol Psychiatry. 2025 Aug 25;31(2):714–25. doi: 10.1038/s41380-025-03158-y (PMC12815654; doi:10.1038/s41380-025-03158-y)
Supplement: Supplementary file 4 — Supplemental Figure 4 [file 41380_2025_3158_MOESM4_ESM.docx]

**
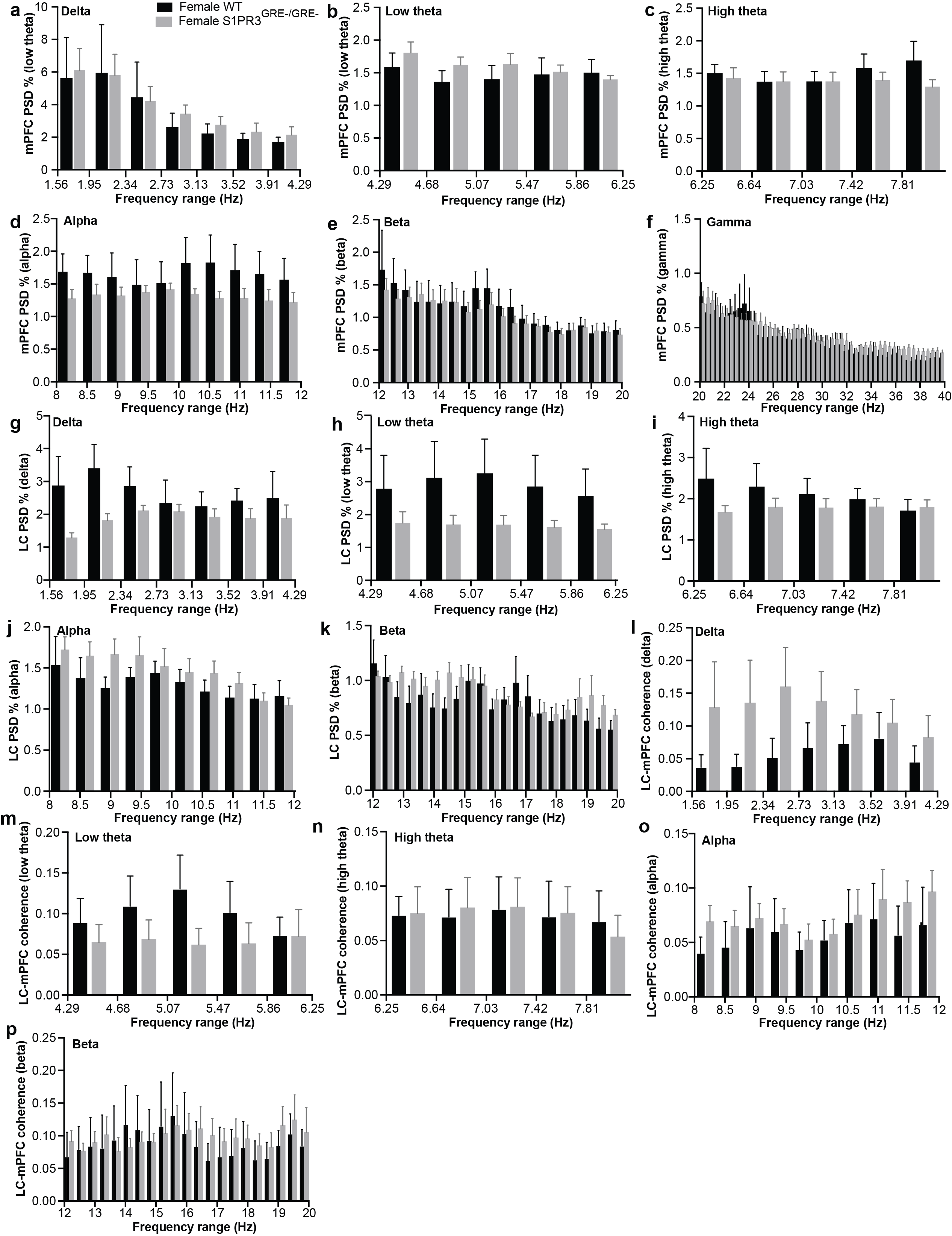
**

**Supplementary Figure 4. mPFC and LC power spectral density percentages and LC-mPFC coherence in non-defeated wild-type and S1PR3^GRE-/GRE-^ females.** Power spectral density percentages in the mPFC in the (**a**) delta, (**b**) low theta, (**c**) high theta, (**d**) alpha, (**e**) beta, and (**f**) gamma frequency ranges. Power spectral density percentages in the LC in the (**g**) delta, (**h**) low theta, (**i**) high theta, (**j**) alpha, and (**k**) beta frequency ranges. LC-mPFC coherence in in the (**l**) delta, (**m**) low theta, (**n**) high theta, (**o**) alpha, and (**p**) beta frequency ranges. For all panels and timepoints, WT mCherry (n=7), S1PR3^GRE-/GRE-^ mCherry (n=6), WT hM4D (n=6), S1PR3^GRE-/GRE-^ hM4D (n=5). Lines represent means ± SEM. Lack of differences assessed by Fisher’s Least Significant Difference Test following 3-way ANOVA.
