# Supplementary material for: A single glucocorticoid response element regulates sociability in a sex-specific manner
Source: Mol Psychiatry. 2025 Aug 25;31(2):714–25. doi: 10.1038/s41380-025-03158-y (PMC12815654; doi:10.1038/s41380-025-03158-y)
Supplement: Supplementary file 5 — Supplemental Table 1 [file 41380_2025_3158_MOESM5_ESM.docx]

**Supplementary Table 1. Raw data values and sample sizes for each panel**

| **Figure** | **Parameter** | **Groups** | **n** | **Raw Avg ± SEM** | **Unit** |
| --- | --- | --- | --- | --- | --- |
| 1b | Enrichment of GR binding to the GRE near S1PR3 | Non-defeated WT males  Defeated WT males | 3  4 | 1 ± 0.339  2.87 ± 0.298 | Normalized fold change (2^-∆∆Ct^) |
| 1d | S1PR3-IR in PL | Non-defeated WT males  Non-defeated S1PR3^GRE-/GRE-^ males  Non-defeated WT females  Non-defeated S1PR3^GRE-/GRE-^ females  Defeated WT males  Defeated S1PR3^GRE-/GRE-^ males  Defeated WT females  Defeated S1PR3^GRE-/GRE-^ females | 9  9  6  7  12  11  6  11 | 10.49 ± 2.201  13.18 ± 2.127  27.15 ± 1.942  9.756 ± 0.951  22.45 ± 5.004  8.806 ± 1.678  34.68 ± 7.603  13.03 ± 0.853 | Arbitrary units (mean pixel intensity) |
| 1e | S1PR3-IR in IL | Non-defeated WT males  Non-defeated S1PR3^GRE-/GRE-^ males  Non-defeated WT females  Non-defeated S1PR3^GRE-/GRE-^ females  Defeated WT males  Defeated S1PR3^GRE-/GRE-^ males  Defeated WT females  Defeated S1PR3^GRE-/GRE-^ females | 9  9  6  7  12  11  6  11 | 10.45 ± 1.439  10.152 ± 1.63  23.041 ± 1.89  8.553 ± 1.024  21.093 ± 3.70  11.660 ± 1.446  26.668 ± 3.37  13.42 ± 1.46 | Arbitrary units (mean pixel intensity) |
| 1f | S1PR3-IR in IL | Non-defeated sham control females  Non-defeated ADX females | 3  6 | 1 ± 0.151  0.4396 ± 0.0815 | Arbitrary units (mean pixel intensity) |
| 1g | S1PR3 mRNA in blood | Non-defeated WT males  Non-defeated S1PR3^GRE-/GRE-^ males  Non-defeated WT females  Non-defeated S1PR3^GRE-/GRE-^ females | 5  4  4  4 | 1 ± 0.467  0.893 ± 0.454  3.226 ± 0.686  0.804 ± 0.294 | Normalized fold change (2^-∆∆Ct^) |
| 2a | Defeat latency | Defeated WT males  Defeated S1PR3^GRE-/GRE-^ males | 12  16 | 669.6 ± 65.05  423.7 ± 54.3 | Time (seconds) |
| 2b | Defeat latency | Defeated WT females  Defeated S1PR3^GRE-/GRE-^ females | 9  11 | 315 ± 48.53  351.7 ± 52.2 | Time (seconds) |
| 2c | Time interacting with stimulus rat | Non-defeated WT males  Non-defeated S1PR3^GRE-/GRE-^ males  Non-defeated WT females  Non-defeated S1PR3^GRE-/GRE-^ females  Defeated WT males  Defeated S1PR3^GRE-/GRE-^ males  Defeated WT females  Defeated S1PR3^GRE-/GRE-^ females | 8  12  8  12  12  16  9  10 | 174.75 ± 7.16  176.255 ± 16.193  168.56 ± 11.67  111.52 ± 6.8  121.57 ± 11.15  70.01 ± 3.14  138.093 ± 9.44  84.717 ± 7.246 | Time (seconds) |
| 3a | Microglia density in PL | Non-defeated WT males  Non-defeated S1PR3^GRE-/GRE-^ males  Non-defeated WT females  Non-defeated S1PR3^GRE-/GRE-^ females  Defeated WT males  Defeated S1PR3^GRE-/GRE-^ males  Defeated WT females  Defeated S1PR3^GRE-/GRE-^ females | 17  9  6  6  12  17  7  11 | 123.73 ± 2.826  119.18 ± 6.218  108.042 ± 4.218  117.58 ± 6.176  115.48 ± 4.623  112.03 ± 4.01  106.171 ± 4.112  107.55 ± 3.159 | Number of IBA1+ cells/mm^2^ |
| 3b | Microglia density in IL | Non-defeated WT males  Non-defeated S1PR3^GRE-/GRE-^ males  Non-defeated WT females  Non-defeated S1PR3^GRE-/GRE-^ females  Defeated WT males  Defeated S1PR3^GRE-/GRE-^ males  Defeated WT females  Defeated S1PR3^GRE-/GRE-^ females | 17  9  6  6  12  17  7  11 | 125.63 ± 2.981  112.89 ± 5.351  104.28 ± 3.761  116.57 ± 6.192  119.291 ± 3.518  138.424 ± 5.243  111.889 ± 8.429  101.486 ± 3.959 | Number of IBA1+ cells/mm^2^ |
| 3d | Monocyte concentration | Non-defeated WT males  Non-defeated S1PR3^GRE-/GRE-^ males  Non-defeated WT females  Non-defeated S1PR3^GRE-/GRE-^ females  Defeated WT males  Defeated S1PR3^GRE-/GRE-^ males  Defeated WT females  Defeated S1PR3^GRE-/GRE-^ females | 8  10  6  8  8  15  9  10 | 178.75 ± 19.034  237.0 ± 31.093  53.333 ± 14.53  166.25 ± 30.469  63.75 ± 6.797  182.0 ± 18.0  163.333 ± 10.408  156.0 ± 19.333 | Number of monocytes/µL |
| 3e | Neutrophil concentration | Non-defeated WT males  Non-defeated S1PR3^GRE-/GRE-^ males  Non-defeated WT females  Non-defeated S1PR3^GRE-/GRE-^ females  Defeated WT males  Defeated S1PR3^GRE-/GRE-^ males  Defeated WT females  Defeated S1PR3^GRE-/GRE-^ females | 8  9  6  9  9  11  7  9 | 856.25 ± 143.787  957.78 ± 83.48  813.333 ± 212.645  687.778 ± 72.03  523.333 ± 117.899  882.94 ± 96.567  690.0 ± 104.232  607.78 ± 56.02 | Number of neutrophils/µL |
| 3f | Lymphocyte concentration | Non-defeated WT males  Non-defeated S1PR3^GRE-/GRE-^ males  Non-defeated WT females  Non-defeated S1PR3^GRE-/GRE-^ females  Defeated WT males  Defeated S1PR3^GRE-/GRE-^ males  Defeated WT females  Defeated S1PR3^GRE-/GRE-^ females | 8  9  6  9  9  11  7  9 | 3807.5 ± 507.89  4264.0 ± 428.74  2380.0 ± 319.91  3060.0 ± 382.16  2662.2 ± 573.83  3510.6 ± 373.41  3667.5 ± 271.88  2549.0 ± 269.6 | Number of lymphocytes /µL |
| 3g | Neutrophil to lymphocyte ratio | Non-defeated WT males  Non-defeated S1PR3^GRE-/GRE-^ males  Non-defeated WT females  Non-defeated S1PR3^GRE-/GRE-^ females  Defeated WT males  Defeated S1PR3^GRE-/GRE-^ males  Defeated WT females  Defeated S1PR3^GRE-/GRE-^ females | 8  9  6  9  9  11  7  9 | 0.226 ± 0.029  0.190 ± 0.013  0.366 ± 0.094  0.243 ± 0.030  0.180 ± 0.028  0.335 ± 0.024  0.202 ± 0.028  0.255 ± 0.033 | Ratio of neutrophil concentration divided by lymphocyte concentration |
| 4c, Supp. Fig. 2a | LC-mPFC coherence (delta, 1.5-4 Hz) | Baseline day 1 WT mCherry  Post-defeat day 1 WT mCherry  Baseline day 7 WT mCherry  Post-defeat day 7 WT mCherry  Baseline day 1 WT hM4D  Post-defeat day 1 WT hM4D  Baseline day 7 WT hM4D  Post-defeat day 7 WT hM4D  Baseline day 1 S1PR3^GRE-/GRE-^ mCherry  Post-defeat day 1 S1PR3^GRE-/GRE-^ mCherry  Baseline day 7 S1PR3^GRE-/GRE-^ mCherry  Post-defeat day 7 S1PR3^GRE-/GRE-^ mCherry  Baseline day 1 S1PR3^GRE-/GRE-^ hM4D  Post-defeat day 1 S1PR3^GRE-/GRE-^ hM4D  Baseline day 7 S1PR3^GRE-/GRE-^ hM4D  Post-defeat day 7 S1PR3^GRE-/GRE-^ hM4D | 7  7  7  7  6  6  6  6  6  6  6  6  5  5  5  5 | 0.096 ± 0.051  0.121 ± 0.083  0.029 ± 0.012  0.040 ± 0.017  0.166 ± 0.048  0.061 ± 0.029  0.226 ± 0.097  0.150 ± 0.090  0.057 ± 0.025  0.289 ± 0.165  0.064 ± 0.050  0.438 ± 0.179  0.037 ± 0.19  0.129 ± 0.047  0.049 ± 0.038  0.017 ± 0.012 | Arbitrary units |
| 4d, Supp. Fig. 2b | LC-mPFC coherence (low theta, 4-6 Hz) | Baseline day 1 WT mCherry  Post-defeat day 1 WT mCherry  Baseline day 7 WT mCherry  Post-defeat day 7 WT mCherry  Baseline day 1 WT hM4D  Post-defeat day 1 WT hM4D  Baseline day 7 WT hM4D  Post-defeat day 7 WT hM4D  Baseline day 1 S1PR3^GRE-/GRE-^ mCherry  Post-defeat day 1 S1PR3^GRE-/GRE-^ mCherry  Baseline day 7 S1PR3^GRE-/GRE-^ mCherry  Post-defeat day 7 S1PR3^GRE-/GRE-^ mCherry  Baseline day 1 S1PR3^GRE-/GRE-^ hM4D  Post-defeat day 1 S1PR3^GRE-/GRE-^ hM4D  Baseline day 7 S1PR3^GRE-/GRE-^ hM4D  Post-defeat day 7 S1PR3^GRE-/GRE-^ hM4D | 7  7  7  7  6  6  6  6  6  6  6  6  5  5  5  5 | 0.040 ± 0.012  0.046 ± 0.023  0.071 ± 0.043  0.044 ± 0.012  0.160 ± 0.036  0.025 ± 0.012  0.214 ± 0.078  0.095 ± 0.064  0.044 ± 0.022  0.206 ± 0.126  0.103 ± 0.085  0.420 ± 0.181  0.035 ± 0.016  0.102 ± 0.016  0.042 ± 0.033  0.022 ± 0.014 | Arbitrary units |
| 4e, Supp. Fig. 2c | LC-mPFC coherence (high theta, 6-8 Hz) | Baseline day 1 WT mCherry  Post-defeat day 1 WT mCherry  Baseline day 7 WT mCherry  Post-defeat day 7 WT mCherry  Baseline day 1 WT hM4D  Post-defeat day 1 WT hM4D  Baseline day 7 WT hM4D  Post-defeat day 7 WT hM4D  Baseline day 1 S1PR3^GRE-/GRE-^ mCherry  Post-defeat day 1 S1PR3^GRE-/GRE-^ mCherry  Baseline day 7 S1PR3^GRE-/GRE-^ mCherry  Post-defeat day 7 S1PR3^GRE-/GRE-^ mCherry  Baseline day 1 S1PR3^GRE-/GRE-^ hM4D  Post-defeat day 1 S1PR3^GRE-/GRE-^ hM4D  Baseline day 7 S1PR3^GRE-/GRE-^ hM4D  Post-defeat day 7 S1PR3^GRE-/GRE-^ hM4D | 7  7  7  7  6  6  6  6  6  6  6  6  5  5  5  5 | 0.018 ± 0.004  0.294 ± 0.062  0.039 ± 0.012  0.069 ± 0.017  0.046 ± 0.025  0.020 ± 0.012  0.204 ± 0.071  0.115 ± 0.041  0.031 ± 0.014  0.195 ± 0.115  0.099 ± 0.077  0.471 ± 0.143  0.058 ± 0.033  0.098 ± 0.039  0.051 ± 0.035  0.021 ± 0.012 | Arbitrary units |
| 4f, Supp. Fig. 2d | LC-mPFC coherence (alpha, 8-12 Hz) | Baseline day 1 WT mCherry  Post-defeat day 1 WT mCherry  Baseline day 7 WT mCherry  Post-defeat day 7 WT mCherry  Baseline day 1 WT hM4D  Post-defeat day 1 WT hM4D  Baseline day 7 WT hM4D  Post-defeat day 7 WT hM4D  Baseline day 1 S1PR3^GRE-/GRE-^ mCherry  Post-defeat day 1 S1PR3^GRE-/GRE-^ mCherry  Baseline day 7 S1PR3^GRE-/GRE-^ mCherry  Post-defeat day 7 S1PR3^GRE-/GRE-^ mCherry  Baseline day 1 S1PR3^GRE-/GRE-^ hM4D  Post-defeat day 1 S1PR3^GRE-/GRE-^ hM4D  Baseline day 7 S1PR3^GRE-/GRE-^ hM4D  Post-defeat day 7 S1PR3^GRE-/GRE-^ hM4D | 7  7  7  7  6  6  6  6  6  6  6  6  5  5  5  5 | 0.031 ± 0.010  0.194 ± 0.104  0.047 ± 0.017  0.081 ± 0.027  0.173 ± 0.052  0.016 ± 0.003  0.226 ± 0.101  0.072 ± 0.020  0.037 ± 0.014  0.101 ± 0.038  0.166 ± 0.093  0.446 ± 0.143  0.061 ± 0.021  0.152 ± 0.069  0.052 ± 0.032  0.026 ± 0.013 | Arbitrary units |
| 4g, Supp. Fig. 2e | LC-mPFC coherence (beta, 12-20 Hz) | Baseline day 1 WT mCherry  Post-defeat day 1 WT mCherry  Baseline day 7 WT mCherry  Post-defeat day 7 WT mCherry  Baseline day 1 WT hM4D  Post-defeat day 1 WT hM4D  Baseline day 7 WT hM4D  Post-defeat day 7 WT hM4D  Baseline day 1 S1PR3^GRE-/GRE-^ mCherry  Post-defeat day 1 S1PR3^GRE-/GRE-^ mCherry  Baseline day 7 S1PR3^GRE-/GRE-^ mCherry  Post-defeat day 7 S1PR3^GRE-/GRE-^ mCherry  Baseline day 1 S1PR3^GRE-/GRE-^ hM4D  Post-defeat day 1 S1PR3^GRE-/GRE-^ hM4D  Baseline day 7 S1PR3^GRE-/GRE-^ hM4D  Post-defeat day 7 S1PR3^GRE-/GRE-^ hM4D | 7  7  7  7  6  6  6  6  6  6  6  6  5  5  5  5 | 0.050 ± 0.011  0.136 ± 0.088  0.072 ± 0.031  0.062 ± 0.022  0.114 ± 0.033  0.030 ± 0.005  0.247 ± 0.108  0.119 ± 0.039  0.051 ± 0.007  0.123 ± 0.043  0.188 ± 0.108  0.475 ± 0.118  0.061 ± 0.028  0.183 ± 0.072  0.081 ± 0.049  0.040 ± 0.013 | Arbitrary units |
| 4h, Supp. Fig. 2f | LC-mPFC coherence (gamma, 20-40 Hz) | Baseline day 1 WT mCherry  Post-defeat day 1 WT mCherry  Baseline day 7 WT mCherry  Post-defeat day 7 WT mCherry  Baseline day 1 WT hM4D  Post-defeat day 1 WT hM4D  Baseline day 7 WT hM4D  Post-defeat day 7 WT hM4D  Baseline day 1 S1PR3^GRE-/GRE-^ mCherry  Post-defeat day 1 S1PR3^GRE-/GRE-^ mCherry  Baseline day 7 S1PR3^GRE-/GRE-^ mCherry  Post-defeat day 7 S1PR3^GRE-/GRE-^ mCherry  Baseline day 1 S1PR3^GRE-/GRE-^ hM4D  Post-defeat day 1 S1PR3^GRE-/GRE-^ hM4D  Baseline day 7 S1PR3^GRE-/GRE-^ hM4D  Post-defeat day 7 S1PR3^GRE-/GRE-^ hM4D | 7  7  7  7  6  6  6  6  6  6  6  6  5  5  5  5 | 0.091 ± 0.027  0.152 ± 0.100  0.159 ± 0.065  0.154 ± 0.031  0.161 ± 0.026  0.054 ± 0.019  0.229 ± 0.096  0.132 ± 0.028  0.134 ± 0.040  0.293 ± 0.066  0.231 ± 0.068  0.620 ± 0.134  0.068 ± 0.024  0.257 ± 0.057  0.171 ± 0.096  0.052 ± 0.019 | Arbitrary units |
| 4i | Time interacting with stimulus rat | 0-5 min WT mCherry  0-5 min WT hM4D  0-5 min S1PR3^GRE-/GRE-^ mCherry  0-5 min S1PR3^GRE-/GRE-^ hM4D  5-10 min WT mCherry  5-10 min WT hM4D  5-10 min S1PR3^GRE-/GRE-^ mCherry  10-15 min S1PR3^GRE-/GRE-^ hM4D  10-15 min WT mCherry  10-15 min WT hM4D  10-15 min S1PR3^GRE-/GRE-^ mCherry  10-15 min S1PR3^GRE-/GRE-^ hM4D | 7  6  6  5  7  6  6  5  7  6  6  5 | 26.223 ± 1.663  44.410 ± 5.846  10.118 ± 1.869  25.188 ± 3.001  32.373 ± 4.265  55.322 ± 2.542  20.492 ± 2.463  38.398 ± 7.665  24.374 ± 2.282  53.622 ±5.663  20.046 ± 3.97  43.000 ± 3.275 | Time (sec) |
| 4j | Mean LC-mPFC coherence across gamma frequency range (20-40 Hz) | WT female  S1PR3^GRE-/GRE-^ female | 5  7 | 0.078 ± 0.013  0.180 ± 0.035 | Arbitrary units |
| 4k | Mean LC power spectral density percentage across gamma frequency range (20-40 Hz) | WT female  S1PR3^GRE-/GRE-^ female | 5  7 | 0.332 ± 0.043  0.482 ± 0.042 | Percentage |
