# Supplementary material for: A single glucocorticoid response element regulates sociability in a sex-specific manner
Source: Mol Psychiatry. 2025 Aug 25;31(2):714–25. doi: 10.1038/s41380-025-03158-y (PMC12815654; doi:10.1038/s41380-025-03158-y)
Supplement: Supplementary file 6 — Supplemental Table 2 [file 41380_2025_3158_MOESM6_ESM.docx]

**Supplementary Table 2. Statistical analyses for each panel**

| **Fig.** | **Parameter** | **Groups** | **Test used** | **Values** | **p-value** |
| --- | --- | --- | --- | --- | --- |
| 1b | Enrichment of GR binding to the GRE near S1PR3 | Non-defeated WT males  Defeated WT males | Student’s t-test | t_5_ = 3.958 | 0.0108 |
| 1d | S1PR3-IR in PL | Non-defeated WT males  Non-defeated S1PR3^GRE-/GRE-^ males  Non-defeated WT females  Non-defeated S1PR3^GRE-/GRE-^ females  Defeated WT males  Defeated S1PR3^GRE-/GRE-^ males  Defeated WT females  Defeated S1PR3^GRE-/GRE-^ females | 3-way ANOVA | Genotype F_1,62_ = 25.14  Defeat F_1,62_ = 3.402  Sex F_1,62_ = 8.869  Genotype x Defeat F_1,62_ = 4.264  Genotype x Sex F_1,62_ = 7.939  Defeat x Sex F_1,62_ = 0.1036  Genotype x Defeat x Sex F_1,62_ = 1.466 | <0.0001  0.0699  0.0041  0.0431  0.0065  0.7486  0.2306 |
|  |  | WT male no defeat vs. WT female no defeat  WT male no defeat vs. WT female defeat  WT female no defeat vs. S1PR3^GRE-/GRE-^ male defeat  WT male defeat vs. S1PR3^GRE-/GRE-^ male defeat  WT female defeat vs. S1PR3^GRE-/GRE-^ male no defeat  WT female defeat vs. S1PR3^GRE-/GRE-^ female no defeat  WT female defeat vs. S1PR3^GRE-/GRE-^ male defeat  WT female defeat vs. S1PR3^GRE-/GRE-^ female defeat | Tukey’s multiple comparisons |  | 0.0482  0.0006  0.0137  0.0364  0.0033  0.0015  <0.0001  0.0018 |
| 1e | S1PR3-IR in IL | Non-defeated WT males  Non-defeated S1PR3^GRE-/GRE-^ males  Non-defeated WT females  Non-defeated S1PR3^GRE-/GRE-^ females  Defeated WT males  Defeated S1PR3^GRE-/GRE-^ males  Defeated WT females  Defeated S1PR3^GRE-/GRE-^ females | 3-way ANOVA | Genotype F_1,63_ = 29.62  Defeat F_1,63_ = 8.993  Sex F_1,63_ = 7.088  Genotype x Defeat F_1,63_ = 1.316  Genotype x Sex F_1,63_ = 6.842  Defeat x Sex F_1,63_ = 0.2827  Genotype x Defeat x Sex F_1,63_ = 2.272 | <0.0001  0.0039  0.0098  0.2557  0.0111  0.5968  0.1368 |
|  |  | WT male no defeat vs. WT female no defeat  WT male no defeat vs. WT male defeat  WT male no defeat vs. WT female defeat  WT female no defeat vs. S1PR3^GRE-/GRE-^ male no defeat  WT female no defeat vs. S1PR3^GRE-/GRE-^ female no defeat  WT female no defeat vs. S1PR3^GRE-/GRE-^ male defeat  WT male defeat vs. S1PR3^GRE-/GRE-^ male no defeat  WT male defeat vs. S1PR3^GRE-/GRE-^ female no defeat  WT male defeat vs. S1PR3^GRE-/GRE-^ male defeat  WT female defeat vs. S1PR3^GRE-/GRE-^ male no defeat  WT female defeat vs. S1PR3^GRE-/GRE-^ female no defeat  WT female defeat vs. S1PR3^GRE-/GRE-^ male defeat  WT female defeat vs. S1PR3^GRE-/GRE-^ female defeat | Tukey’s multiple comparisons |  | 0.0239  0.0217  0.0011  0.0190  0.0097  0.0424  0.0164  0.0085  0.0397  0.0008  0.0005  0.0020  0.0095 |
| 1f | S1PR3-IR in IL | Non-defeated sham control female  Non-defeated ADX female | Student’s t-test | t_7_ = 3.06 | 0.0085 |
| 1g | S1PR3 mRNA in blood | WT males  S1PR3^GRE-/GRE-^ males  WT females  S1PR3^GRE-/GRE-^ females | 2-way ANOVA | Genotype F_1,13_ = 6.543  Sex F_1,13_ = 4.617  Interaction F_1,13_ = 5.424 | 0.0245  0.0511  0.0366 |
|  |  | WT male vs. WT female  WT female vs. S1PR3^GRE-/GRE-^ male  WT female vs. S1PR3^GRE-/GRE-^ female | Tukey’s multiple comparisons |  | 0.0282  0.0292  0.0233 |
| 2a | Defeat latency | WT males  S1PR3^GRE-/GRE-^ males | Student’s t-test | t_13_ = 2.852 | 0.0136 |
| 2b | Defeat latency | Defeated WT females  Defeated S1PR3^GRE-/GRE-^ females | Student’s t-test | t_18_ = 0.5064 | 0.6187 |
| 2c | Time interacting with stimulus rat | Non-defeated WT males  Non-defeated S1PR3^GRE-/GRE-^ males  Non-defeated WT females  Non-defeated S1PR3^GRE-/GRE-^ females  Defeated WT males  Defeated S1PR3^GRE-/GRE-^ males  Defeated WT females  Defeated S1PR3^GRE-/GRE-^ females | 3-way ANOVA | Genotype F_1,79_ = 32.40  Defeat F_1,79_ = 59.08  Sex F_1,79_ = 1.982  Genotype x Defeat F_1,79_ = 3.070  Genotype x Sex F_1,79_ = 4.585  Defeat x Sex F_1,79_ = 13.13  Genotype x Defeat x Sex F_1,79_ = 4.049 | <0.0001  <0.0001  0.1631  0.0836  0.0353  0.0005  0.0476 |
|  |  | WT male no defeat vs. WT male defeat  WT male no defeat vs. S1PR3^GRE-/GRE-^ female no defeat  WT male no defeat vs. S1PR3^GRE-/GRE-^ male defeat  WT male no defeat vs. S1PR3^GRE-/GRE-^ female defeat  WT female no defeat vs. WT male defeat  WT female no defeat vs. S1PR3^GRE-/GRE-^ female no defeat  WT female no defeat vs. S1PR3^GRE-/GRE-^ male defeat  WT female no defeat vs. S1PR3^GRE-/GRE-^ female defeat  WT male defeat vs. S1PR3^GRE-/GRE-^ male no defeat  WT male defeat vs. S1PR3^GRE-/GRE-^ male defeat  WT female defeat vs. S1PR3^GRE-/GRE-^ male defeat  WT female defeat vs. S1PR3^GRE-/GRE-^ female defeat  S1PR3^GRE-/GRE-^ male no defeat vs. S1PR3^GRE-/GRE-^ female no defeat  S1PR3^GRE-/GRE-^ male no defeat vs. S1PR3^GRE-/GRE-^ male defeat  S1PR3^GRE-/GRE-^ male no defeat vs. S1PR3^GRE-/GRE-^ female defeat  S1PR3^GRE-/GRE-^ female no defeat vs. S1PR3^GRE-/GRE-^ male defeat | Tukey’s multiple comparisons |  | 0.0111  0.0011  <0.0001  <0.0001  0.0383  0.0048  <0.0001  <0.0001  0.0018  0.0018  <0.0001  0.0115  0.0001  <0.0001  <0.0001  0.0231 |
| 3a | Microglia density in PL | Non-defeated WT males  Non-defeated S1PR3^GRE-/GRE-^ males  Non-defeated WT females  Non-defeated S1PR3^GRE-/GRE-^ females  Defeated WT males  Defeated S1PR3^GRE-/GRE-^ males  Defeated WT females  Defeated S1PR3^GRE-/GRE-^ females | 3-way ANOVA | Genotype F_1,77_ = 0.048  Defeat F_1,77_ = 4.173  Sex F_1,77_ = 5.409  Genotype x Defeat F_1,77_ = 0.2793  Genotype x Sex F_1,77_ = 2.006  Defeat x Sex F_1,77_ = 0.06811  Genotype x Defeat x Sex F_1,77_ = 0.4808 | 0.8279  0.0445  0.0227  0.5987  0.1607  0.7948  0.4902 |
|  |  | No significant differences among any subgroups | Tukey’s multiple comparisons |  |  |
| 3b | Microglia density in IL | Non-defeated WT males  Non-defeated S1PR3^GRE-/GRE-^ males  Non-defeated WT females  Non-defeated S1PR3^GRE-/GRE-^ females  Defeated WT males  Defeated S1PR3^GRE-/GRE-^ males  Defeated WT females  Defeated S1PR3^GRE-/GRE-^ females | 3-way ANOVA | Genotype F_1,77_ = 0.3115  Defeat F_1,77_ = 0.6243  Sex F_1,77_ = 17.47  Genotype x Defeat F_1,77_ = 0.3828  Genotype x Sex F_1,77_ = 0.0921  Defeat x Sex F_1,77_ = 3.230  Genotype x Defeat x Sex F_1,77_ = 13.53 | 0.5785  0.4320  <0.0001  0.5380  0.7624  0.0764 |
|  |  | WT male no defeat vs. S1PR3^GRE-/GRE-^ female defeat  WT female no defeat vs. S1PR3^GRE-/GRE-^ male defeat  WT male defeat vs. S1PR3^GRE-/GRE-^ male defeat  WT female defeat vs. S1PR3^GRE-/GRE-^ male defeat  S1PR3^GRE-/GRE-^ male no defeat vs. S1PR3^GRE-/GRE-^ male defeat  S1PR3^GRE-/GRE-^ male defeat vs. S1PR3^GRE-/GRE-^ female defeat | Tukey’s multiple comparisons |  | 0.0038  0.0004  0.0457  0.0136  0.0041  <0.0001 |
| 3d | Monocyte concentration | Non-defeated WT males  Non-defeated S1PR3^GRE-/GRE-^ males  Non-defeated WT females  Non-defeated S1PR3^GRE-/GRE-^ females  Defeated WT males  Defeated S1PR3^GRE-/GRE-^ males  Defeated WT females  Defeated S1PR3^GRE-/GRE-^ females | 3-way ANOVA | Genotype F_1,66_ = 20.57  Defeat F_1,66_ = 1.267  Sex F_1,66_ = 3.884  Genotype x Defeat F_1,66_ = 0.9384  Genotype x Sex F_1,66_ = 1.300  Defeat x Sex F_1,66_ = 18.81  Genotype x Defeat x Sex F_1,66_ = 8.399 | <0.0001  0.2628  0.0529  0.3362  0.2583  <0.0001  0.0051 |
|  |  | WT male no defeat vs. WT female no defeat  WT male no defeat vs. WT male defeat  WT female no defeat vs. WT female defeat  WT female no defeat vs. S1PR3^GRE-/GRE-^ male no defeat  WT female no defeat vs. S1PR3^GRE-/GRE-^ female no defeat  WT female no defeat vs. S1PR3^GRE-/GRE-^ male defeat  WT male defeat vs. WT female defeat  WT male defeat vs. S1PR3^GRE-/GRE-^ male no defeat  WT male defeat vs. S1PR3^GRE-/GRE-^ female no defeat  WT male defeat vs. S1PR3^GRE-/GRE-^ male defeat | Tukey’s multiple comparisons |  | 0.0142  0.0157  0.0397  <0.0001  0.0391  0.0027  0.0463  <0.0001  0.0462  0.0022 |
| 3e | Neutrophil concentration | Non-defeated WT males  Non-defeated S1PR3^GRE-/GRE-^ males  Non-defeated WT females  Non-defeated S1PR3^GRE-/GRE-^ females  Defeated WT males  Defeated S1PR3^GRE-/GRE-^ males  Defeated WT females  Defeated S1PR3^GRE-/GRE-^ females | 3-way ANOVA | Genotype F_1,66_ = 1.085  Defeat F_1,66_ = 4.647  Sex F_1,66_ = 2.449  Genotype x Defeat F_1,66_ = 0.4715  Genotype x Sex F_1,66_ = 5.456  Defeat x Sex F_1,66_ = 0.7903  Genotype x Defeat x Sex F_1,66_ = 0.1736 | 0.3015  0.0348  0.1224  0.4947  0.0225  0.3772  0.6782 |
|  |  | WT male defeat vs. S1PR3^GRE-/GRE-^ male no defeat | Tukey’s multiple comparisons |  | 0.0424 |
| 3f | lymphocyte concentration | Non-defeated WT males  Non-defeated S1PR3^GRE-/GRE-^ males  Non-defeated WT females  Non-defeated S1PR3^GRE-/GRE-^ females  Defeated WT males  Defeated S1PR3^GRE-/GRE-^ males  Defeated WT females  Defeated S1PR3^GRE-/GRE-^ females | 3-way ANOVA | Genotype F_1,66_ = 0.532  Defeat F_1,66_ = 0.8928  Sex F_1,66_ = 4.748  Genotype x Defeat F_1,66_ = 1.403  Genotype x Sex F_1,66_ = 2.155  Defeat x Sex F_1,66_ = 5.074  Genotype x Defeat x Sex F_1,66_ = 3.401 | 0.4682  0.3480  0.0327  0.2403  0.1466  0.0274  0.0694 |
|  |  | No significant differences among any subgroups | Tukey’s multiple comparisons |  |  |
| 3g | Neutrophil to lymphocyte ratio | Non-defeated WT males  Non-defeated S1PR3^GRE-/GRE-^ males  Non-defeated WT females  Non-defeated S1PR3^GRE-/GRE-^ females  Defeated WT males  Defeated S1PR3^GRE-/GRE-^ males  Defeated WT females  Defeated S1PR3^GRE-/GRE-^ females | 3-way ANOVA | Genotype F_1,59_ = 0.239  Defeat F_1,59_ = 0.2743  Sex F_1,59_ = 1.805  Genotype x Defeat F_1,59_ = 13.07  Genotype x Sex F_1,59_ = 3.529  Defeat x Sex F_1,59_ = 6.230  Genotype x Defeat x Sex F_1,59_ = 0.0209 | 0.6272  0.6024  0.1843  0.0006  0.0652  0.0154  0.8854 |
|  |  | WT female no defeat vs. WT male defeat  WT female no defeat vs. S1PR3^GRE-/GRE-^ male no defeat  WT male defeat vs. S1PR3^GRE-/GRE-^ male defeat  S1PR3^GRE-/GRE-^ male no defeat vs. S1PR3^GRE-/GRE-^ male defeat | Tukey’s multiple comparisons |  | 0.0265  0.0350  0.0357  0.0474 |
| 4c, Supp. Fig. 2a | LC-mPFC coherence (delta, 1.5-4 Hz) | Baseline day 1 WT mCherry  Post-defeat day 1 WT mCherry  Baseline day 7 WT mCherry  Post-defeat day 7 WT mCherry  Baseline day 1 WT hM4D  Post-defeat day 1 WT hM4D  Baseline day 7 WT hM4D  Post-defeat day 7 WT hM4D  Baseline day 1 S1PR3^GRE-/GRE-^ mCherry  Post-defeat day 1 S1PR3^GRE-/GRE-^ mCherry  Baseline day 7 S1PR3^GRE-/GRE-^ mCherry  Post-defeat day 7 S1PR3^GRE-/GRE-^ mCherry  Baseline day 1 S1PR3^GRE-/GRE-^ hM4D  Post-defeat day 1 S1PR3^GRE-/GRE-^ hM4D  Baseline day 7 S1PR3^GRE-/GRE-^ hM4D  Post-defeat day 7 S1PR3^GRE-/GRE-^ hM4D | 3-way ANOVA | Time F_3,66_ = 0.8569  Genotype F_1,66_ = 0.3470  DREADDs F_1,66_ = 0.8379  Time x Genotype F_3,66_ = 2.066  Time x DREADDs F_3,66_ = 1.941  Genotype x DREADDs F_1,66_ = 8.190  Time x Genotype x DREADDs F_3,66_ = 1.522 | 0.4680  0.5578  0.3633  0.1132  0.1315  0.0056  0.2169 |
|  |  | Baseline day 1 WT hM4D vs. post day 7 S1PR3^GRE-/GRE-^ mCherry  Baseline day 1 WT GFP vs. post day 7 S1PR3^GRE-/GRE-^ mCherry  Baseline day 1 S1PR3^GRE-/GRE-^ hM4D vs. post day 1 S1PR3^GRE-/GRE-^ mCherry  Baseline day 1 S1PR3^GRE-/GRE-^ hM4D vs. post day 7 S1PR3^GRE-/GRE-^ mCherry  Baseline day 1 S1PR3^GRE-/GRE-^ mCherry vs. post day 1 S1PR3^GRE-/GRE-^ mCherry  Baseline day 1 S1PR3^GRE-/GRE-^ mCherry vs. post day 7 S1PR3^GRE-/GRE-^ mCherry  Post day 1 WT hM4D vs. post day 1 S1PR3^GRE-/GRE-^ mCherry  Post day 1 WT hM4D vs. post day 7 S1PR3^GRE-/GRE-^ mCherry  Post day 1 WT mCherry vs. post day 7 S1PR3^GRE-/GRE-^ mCherry  Post day 1 S1PR3^GRE-/GRE-^ hM4D vs. post day 7 S1PR3^GRE-/GRE-^ mCherry  Post day 1 S1PR3^GRE-/GRE-^ mCherry vs. baseline day 7 WT mCherry  Post day 1 S1PR3^GRE-/GRE-^ mCherry vs. post day 7 WT mCherry  Post day 1 S1PR3^GRE-/GRE-^ mCherry vs. post day 7 S1PR3^GRE-/GRE-^ hM4D  Baseline day 7 WT mCherry vs. post day 7 S1PR3^GRE-/GRE-^ mCherry  Baseline day 7 S1PR3^GRE-/GRE-^ hM4D vs. post day 7 S1PR3^GRE-/GRE-^ mCherry  Baseline day 7 S1PR3^GRE-/GRE-^ mCherry vs. post day 7 S1PR3^GRE-/GRE-^ mCherry  Post day 7 WT hM4D vs. post day 7 S1PR3^GRE-/GRE-^ mCherry  Post day 7 WT mCherry vs. post day 7 S1PR3^GRE-/GRE-^ mCherry  Post day 7 S1PR3^GRE-/GRE-^ hM4D vs. post day 7 S1PR3^GRE-/GRE-^ mCherry | Fisher’s LSD |  | 0.0152  0.0019  0.0417  0.0015  0.0468  0.0014  0.0499  0.0015  0.0037  0.0129  0.0203  0.0261  0.0430  0.0004  0.0020  0.0017  0.014  0.0005  0.0021 |
| 4d, Supp. Fig. 2b | LC-mPFC coherence (low theta, 4-6 Hz) | Baseline day 1 WT mCherry  Post-defeat day 1 WT mCherry  Baseline day 7 WT mCherry  Post-defeat day 7 WT mCherry  Baseline day 1 WT hM4D  Post-defeat day 1 WT hM4D  Baseline day 7 WT hM4D  Post-defeat day 7 WT hM4D  Baseline day 1 S1PR3^GRE-/GRE-^ mCherry  Post-defeat day 1 S1PR3^GRE-/GRE-^ mCherry  Baseline day 7 S1PR3^GRE-/GRE-^ mCherry  Post-defeat day 7 S1PR3^GRE-/GRE-^ mCherry  Baseline day 1 S1PR3^GRE-/GRE-^ hM4D  Post-defeat day 1 S1PR3^GRE-/GRE-^ hM4D  Baseline day 7 S1PR3^GRE-/GRE-^ hM4D  Post-defeat day 7 S1PR3^GRE-/GRE-^ hM4D | 3-way ANOVA | Time F_3,66_ = 0.8282  Genotype F_1,66_ = 1.068  DREADDs F_1,66_ = 1.046  Time x Genotype F_3,66_ = 2.891  Time x DREADDs F_3,66_ = 2.342  Genotype x DREADDs F_1,66_ = 10.01  Time x Genotype x DREADDs F_3,66_ = 1.353 | 0.4832  0.3052  0.3103  0.0420  0.0813  0.0024  0.2850 |
|  |  | Baseline day 1 WT hM4D vs. post day 7 S1PR3^GRE-/GRE-^ mCherry  Baseline day 1 WT mCherry vs. post day 7 S1PR3^GRE-/GRE-^ mCherry  Baseline day 1 S1PR3^GRE-/GRE-^ hM4D vs. post day 7 S1PR3^GRE-/GRE-^ mCherry  Baseline day 1 S1PR3^GRE-/GRE-^ mCherry vs. post day 7 S1PR3^GRE-/GRE-^ mCherry  Post day 1 WT hM4D vs. post day 7 S1PR3^GRE-/GRE-^ mCherry  Post day 1 WT mCherry vs. post day 7 S1PR3^GRE-/GRE-^ mCherry  Post day 1 S1PR3^GRE-/GRE-^ hM4D vs. post day 7 S1PR3^GRE-/GRE-^ mCherry  Post day 1 S1PR3^GRE-/GRE-^ mCherry vs. post day 7 S1PR3^GRE-/GRE-^ mCherry  Baseline day 7 WT hM4D vs. post day 7 S1PR3^GRE-/GRE-^ mCherry  Baseline day 7 WT mCherry vs. post day 7 S1PR3^GRE-/GRE-^ mCherry  Baseline day 7 S1PR3^GRE-/GRE-^ hM4D vs. post day 7 S1PR3^GRE-/GRE-^ mCherry  Baseline day 7 S1PR3^GRE-/GRE-^ mCherry vs. post day 7 S1PR3^GRE-/GRE-^ mCherry  Post day 7 WT hM4D vs. post day 7 S1PR3^GRE-/GRE-^ mCherry  Post day 7 WT mCherry vs. post day 7 S1PR3^GRE-/GRE-^ mCherry  Post day 7 S1PR3^GRE-/GRE-^ hM4D vs. post day 7 S1PR3^GRE-/GRE-^ mCherry | Fisher’s LSD |  | 0.0058  <0.0001  0.0003  0.0002  <0.0001  0.0001  0.0025  0.0278  0.0338  0.0003  0.0004  0.0014  0.0011  0.0001  0.0006 |
| 4e, Supp. Fig. 2c | LC-mPFC coherence (high theta, 6-8 Hz) | Baseline day 1 WT mCherry  Post-defeat day 1 WT mCherry  Baseline day 7 WT mCherry  Post-defeat day 7 WT mCherry  Baseline day 1 WT hM4D  Post-defeat day 1 WT hM4D  Baseline day 7 WT hM4D  Post-defeat day 7 WT hM4D  Baseline day 1 S1PR3^GRE-/GRE-^ mCherry  Post-defeat day 1 S1PR3^GRE-/GRE-^ mCherry  Baseline day 7 S1PR3^GRE-/GRE-^ mCherry  Post-defeat day 7 S1PR3^GRE-/GRE-^ mCherry  Baseline day 1 S1PR3^GRE-/GRE-^ hM4D  Post-defeat day 1 S1PR3^GRE-/GRE-^ hM4D  Baseline day 7 S1PR3^GRE-/GRE-^ hM4D  Post-defeat day 7 S1PR3^GRE-/GRE-^ hM4D | 3-way ANOVA | Time F_3,66_ = 3.90  Genotype F_1,66_ = 0.842  DREADDs F_1,66_ = 6.245  Time x Genotype F_3,66_ = 1.979  Time x DREADDs F_3,66_ = 5.159  Genotype x DREADDs F_1,66_ = 4.892  Time x Genotype x DREADDs F_3,66_ = 5.573 | 0.0126  0.3621  0.0150  0.1256  0.0029  0.0305  0.0018 |
|  |  | Baseline day 1 WT hM4D vs. post day 1 WT mCherry  Baseline day 1 WT hM4D vs. post day 7 S1PR3^GRE-/GRE-^ mCherry  Baseline day 1 WT mCherry vs. post day 1 WT mCherry  Baseline day 1 WT mCherry vs. post day 1 S1PR3^GRE-/GRE-^ mCherry  Baseline day 1 WT mCherry vs. baseline day 7 WT hM4D  Baseline day 1 WT mCherry vs. post day 7 S1PR3^GRE-/GRE-^ mCherry  Baseline day 1 S1PR3^GRE-/GRE-^ hM4D vs. post day 1 WT mCherry  Baseline day 1 S1PR3^GRE-/GRE-^ hM4D vs. post day 7 S1PR3^GRE-/GRE-^ mCherry  Baseline day 1 S1PR3^GRE-/GRE-^ mCherry vs. post day 1 WT mCherry  Baseline day 1 S1PR3^GRE-/GRE-^ mCherry vs. baseline day 7 WT hM4D  Baseline day 1 S1PR3^GRE-/GRE-^ mCherry vs. post day 7 S1PR3^GRE-/GRE-^ mCherry  Post day 1 WT hM4D vs. post day 1 WT mCherry  Post day 1 WT hM4D vs. post day 1 S1PR3^GRE-/GRE-^ mCherry  Post day 1 WT hM4D vs. baseline day 7 WT hM4D  Post day 1 WT hM4D vs. post day 7 S1PR3^GRE-/GRE-^ mCherry  Post day 1 WT mCherry vs. post day 1 S1PR3^GRE-/GRE-^ hM4D  Post day 1 WT mCherry vs. baseline day 7 WT mCherry  Post day 1 WT mCherry vs. baseline day 7 S1PR3^GRE-/GRE-^ hM4D  Post day 1 WT mCherry vs. baseline day 7 S1PR3^GRE-/GRE-^ mCherry  Post day 1 WT mCherry vs. post day 7 WT hM4D  Post day 1 WT mCherry vs. post day 7 WT mCherry  Post day 1 WT mCherry vs. post day 7 S1PR3^GRE-/GRE-^ hM4D  Post day 1 WT mCherry vs. post day 7 S1PR3^GRE-/GRE-^ mCherry  Post day 1 S1PR3^GRE-/GRE-^ hM4D vs. post day 7 S1PR3^GRE-/GRE-^ mCherry  Post day 1 S1PR3^GRE-/GRE-^ mCherry vs. post day 7 S1PR3^GRE-/GRE-^ mCherry  Baseline day 7 WT hM4D vs. baseline day 7 WT mCherry  Baseline day 7 WT hM4D vs. post day 7 S1PR3^GRE-/GRE-^ mCherry  Baseline day 7 WT mCherry vs. post day 7 S1PR3^GRE-/GRE-^ mCherry  Baseline day 7 S1PR3^GRE-/GRE-^ hM4D vs. post day 7 S1PR3^GRE-/GRE-^ mCherry  Baseline day 7 S1PR3^GRE-/GRE-^ mCherry vs. post day 7 S1PR3^GRE-/GRE-^ mCherry  Post day 7 WT hM4D vs. post day 7 S1PR3^GRE-/GRE-^ mCherry  Post day 7 WT mCherry vs. post day 7 S1PR3^GRE-/GRE-^ mCherry  Post day 7 S1PR3^GRE-/GRE-^ hM4D vs. post day 7 S1PR3^GRE-/GRE-^ mCherry | Fisher’s LSD |  | 0.0014  <0.0001  0.0002  0.0263  0.0201  <0.0001  0.0063  <0.0001  0.0013  0.0453  <0.0001  0.0008  0.0419  0.0332  <0.0001  0.0222  0.0010  0.0051  0.0152  0.0253  0.0035  0.0042  0.0266  <0.0001  0.0017  0.0456  0.0023  <0.0001  <0.0001  <0.0001  <0.0001  <0.0001  <0.0001 |
| 4f, Supp. Fig. 2d | LC-mPFC coherence (alpha, 8-12 Hz) | Baseline day 1 WT mCherry  Post-defeat day 1 WT mCherry  Baseline day 7 WT mCherry  Post-defeat day 7 WT mCherry  Baseline day 1 WT hM4D  Post-defeat day 1 WT hM4D  Baseline day 7 WT hM4D  Post-defeat day 7 WT hM4D  Baseline day 1 S1PR3^GRE-/GRE-^ mCherry  Post-defeat day 1 S1PR3^GRE-/GRE-^ mCherry  Baseline day 7 S1PR3^GRE-/GRE-^ mCherry  Post-defeat day 7 S1PR3^GRE-/GRE-^ mCherry  Baseline day 1 S1PR3^GRE-/GRE-^ hM4D  Post-defeat day 1 S1PR3^GRE-/GRE-^ hM4D  Baseline day 7 S1PR3^GRE-/GRE-^ hM4D  Post-defeat day 7 S1PR3^GRE-/GRE-^ hM4D |  | Time F_3,66_ = 1.033  Genotype F_1,66_ = 0.592  DREADDs F_1,66_ = 1.555  Time x Genotype F_3,66_ = 2.037  Time x DREADDs F_3,66_ = 3.923  Genotype x DREADDs F_1,66_ = 5.191  Time x Genotype x DREADDs F_3,66_ = 4.491 | 0.3839  0.4445  0.2168  0.1174  0.0123  0.0260  0.0063 |
|  |  | Baseline day 1 WT hM4D vs. post day 7 S1PR3^GRE-/GRE-^ mCherry  Baseline day 1 WT mCherry vs. post day 1 WT mCherry  Baseline day 1 WT mCherry vs. baseline day 7 WT hM4D  Baseline day 1 WT mCherry vs. post day 7 S1PR3^GRE-/GRE-^ mCherry  Baseline day 1 S1PR3^GRE-/GRE-^ hM4D vs. post day 7 S1PR3^GRE-/GRE-^ mCherry  Baseline day 1 S1PR3^GRE-/GRE-^ mCherry vs. baseline day 7 WT hM4D  Baseline day 1 S1PR3^GRE-/GRE-^ mCherry vs. post day 7 S1PR3^GRE-/GRE-^ mCherry  Post day 1 WT hM4D vs. post day 1 WT mCherry  Post day 1 WT hM4D vs. baseline day 7 WT hM4D  Post day 1 WT hM4D vs. post day 7 S1PR3^GRE-/GRE-^ mCherry  Post day 1 WT mCherry vs. post day 7 S1PR3^GRE-/GRE-^ mCherry  Post day 1 S1PR3^GRE-/GRE-^ hM4D vs. post day 7 S1PR3^GRE-/GRE-^ mCherry  Baseline day 7 WT hM4D vs. baseline day 7 WT mCherry  Baseline day 7 WT hM4D vs. post day 7 S1PR3^GRE-/GRE-^ mCherry  Baseline day 7 WT mCherry vs. post day 7 S1PR3^GRE-/GRE-^ mCherry  Baseline day 7 S1PR3^GRE-/GRE-^ hM4D vs. post day 7 S1PR3^GRE-/GRE-^ mCherry  Baseline day 7 S1PR3^GRE-/GRE-^ mCherry vs. post day 7 S1PR3^GRE-/GRE-^ mCherry  Post day 7 WT hM4D vs. post day 7 S1PR3^GRE-/GRE-^ mCherry  Post day 7 WT mCherry vs. post day 7 S1PR3^GRE-/GRE-^ mCherry  Post day 7 S1PR3^GRE-/GRE-^ hM4D vs. post day 7 S1PR3^GRE-/GRE-^ mCherry | Fisher’s LSD |  | 0.0026  0.0457  0.0236  <0.0001  0.0002  0.0409  <0.0001  0.0441  0.0236  <0.0001  0.0052  0.0033  0.0431  0.0185  <0.0001  0.0001  0.0030  0.0001  <0.0001  0.0002 |
| 4g, Supp. Fig. 2e | LC-mPFC coherence (beta, 12-20 Hz) | Baseline day 1 WT mCherry  Post-defeat day 1 WT mCherry  Baseline day 7 WT mCherry  Post-defeat day 7 WT mCherry  Baseline day 1 WT hM4D  Post-defeat day 1 WT hM4D  Baseline day 7 WT hM4D  Post-defeat day 7 WT hM4D  Baseline day 1 S1PR3^GRE-/GRE-^ mCherry  Post-defeat day 1 S1PR3^GRE-/GRE-^ mCherry  Baseline day 7 S1PR3^GRE-/GRE-^ mCherry  Post-defeat day 7 S1PR3^GRE-/GRE-^ mCherry  Baseline day 1 S1PR3^GRE-/GRE-^ hM4D  Post-defeat day 1 S1PR3^GRE-/GRE-^ hM4D  Baseline day 7 S1PR3^GRE-/GRE-^ hM4D  Post-defeat day 7 S1PR3^GRE-/GRE-^ hM4D | 3-way ANOVA | Time F_3,66_ = 1.977  Genotype F_1,66_ = 2.130  DREADDs F_1,66_ = 1.203  Time x Genotype F_3,66_ = 1.997  Time x DREADDs F_3,66_ = 2.587  Genotype x DREADDs F_1,66_ = 6.696  Time x Genotype x DREADDs F_3,66_ = 4.791 | 0.1259  0.1492  0.2767  0.1229  0.0604  0.0119  0.0044 |
|  |  | Baseline day 1 WT hM4D vs. post day 7 S1PR3^GRE-/GRE-^ mCherry  Baseline day 1 WT mCherry vs. baseline day 7 WT hM4D  Baseline day 1 WT mCherry vs. post day 7 S1PR3^GRE-/GRE-^ mCherry  Baseline day 1 S1PR3^GRE-/GRE-^ hM4D vs. post day 7 S1PR3^GRE-/GRE-^ mCherry  Baseline day 1 S1PR3^GRE-/GRE-^ mCherry vs. baseline day 7 WT hM4D  Baseline day1 S1PR3^GRE-/GRE-^ mCherry vs. post day 7 S1PR3^GRE-/GRE-^ mCherry  Post day 1 WT hM4D vs. baseline day 7 WT hM4D  Post day 1 WT hM4D vs. post day 7 S1PR3^GRE-/GRE-^ mCherry  Post day 1 WT mCherry vs. post day 7 S1PR3^GRE-/GRE-^ mCherry  Post day 1 S1PR3^GRE-/GRE-^ hM4D vs. post day 7 S1PR3^GRE-/GRE-^ mCherry  Post day 1 S1PR3^GRE-/GRE-^ mCherry vs. post day 7 S1PR3^GRE-/GRE-^ mCherry  Baseline day 7 WT hM4D vs. baseline day 7 WT mCherry  Baseline day 7 WT hM4D vs. post day 7 WT mCherry  Baseline day 7 WT hM4D vs. post day 7 S1PR3^GRE-/GRE-^ hM4D  Baseline day 7 WT hM4D vs. post day 7 S1PR3^GRE-/GRE-^ mCherry  Baseline day 7 WT mCherry vs. post day 7 S1PR3^GRE-/GRE-^ mCherry  Baseline day 7 S1PR3^GRE-/GRE-^ hM4D vs. post day 7 S1PR3^GRE-/GRE-^ mCherry  Baseline day 7 S1PR3^GRE-/GRE-^ mCherry vs. post day 7 S1PR3^GRE-/GRE-^ mCherry  Post day 7 WT hM4D vs. post day 7 S1PR3^GRE-/GRE-^ mCherry  Post day 7 WT mCherry vs. post day 7 S1PR3^GRE-/GRE-^ mCherry  Post day 7 S1PR3^GRE-/GRE-^ hM4D vs. post day 7 S1PR3^GRE-/GRE-^ mCherry | Fisher’s LSD |  | <0.0001  0.0204  <0.0001  <0.0001  0.0322  <0.0001  0.0184  <0.0001  0.0001  0.0031  0.0002  0.0454  0.0342  0.0499  0.0133  <0.0001  <0.0001  0.0021  0.0002  <0.0001  <0.0001 |
| 4h, Supp. Fig. 2f | LC-mPFC coherence (gamma, 20-40 Hz) | Baseline day 1 WT mCherry  Post-defeat day 1 WT mCherry  Baseline day 7 WT mCherry  Post-defeat day 7 WT mCherry  Baseline day 1 WT hM4D  Post-defeat day 1 WT hM4D  Baseline day 7 WT hM4D  Post-defeat day 7 WT hM4D  Baseline day 1 S1PR3^GRE-/GRE-^ mCherry  Post-defeat day 1 S1PR3^GRE-/GRE-^ mCherry  Baseline day 7 S1PR3^GRE-/GRE-^ mCherry  Post-defeat day 7 S1PR3^GRE-/GRE-^ mCherry  Baseline day 1 S1PR3^GRE-/GRE-^ hM4D  Post-defeat day 1 S1PR3^GRE-/GRE-^ hM4D  Baseline day 7 S1PR3^GRE-/GRE-^ hM4D  Post-defeat day 7 S1PR3^GRE-/GRE-^ hM4D | 3-way ANOVA | Time F_3,66_ = 2.325  Genotype F_1,66_ = 4.71  DREADDs F_1,66_ = 4.983  Time x Genotype F_3,66_ = 2.134  Time x DREADDs F_3,66_ = 4.124  Genotype x DREADDs F_1,66_ = 5.654  Time x Genotype x DREADDs F_3,66_ = 4.099 | 0.0829  0.0336  0.0290  0.1043  0.0098  0.0203  0.0099 |
|  |  | Baseline day 1 WT hM4D vs. post day 7 S1PR3^GRE-/GRE-^ mCherry  Baseline day 1 WT mCherry vs. post day 7 S1PR3^GRE-/GRE-^ mCherry  Baseline day 1 S1PR3^GRE-/GRE-^ hM4D vs. post day 7 S1PR3^GRE-/GRE-^ mCherry  Baseline day 1 S1PR3^GRE-/GRE-^ mCherry vs. post day 7 S1PR3^GRE-/GRE-^ mCherry  Post day 1 WT hM4D vs. post day 7 S1PR3^GRE-/GRE-^ mCherry  Post day 1 WT mCherry vs. post day 7 S1PR3^GRE-/GRE-^ mCherry  Post day 1 S1PR3^GRE-/GRE-^ hM4D vs. post day 7 S1PR3^GRE-/GRE-^ mCherry  Post day 1 S1PR3^GRE-/GRE-^ mCherry vs. post day 7 S1PR3^GRE-/GRE-^ mCherry  Baseline day 7 WT hM4D vs. post day 7 S1PR3^GRE-/GRE-^ mCherry  Baseline day 7 WT mCherry vs. post day 7 S1PR3^GRE-/GRE-^ mCherry  Baseline day 7 S1PR3^GRE-/GRE-^hM4D vs. post day 7 S1PR3^GRE-/GRE-^ mCherry  Baseline day 7 S1PR3^GRE-/GRE-^ mCherry vs. post day 7 S1PR3^GRE-/GRE-^ mCherry  Post day 7 WT hM4D vs. post day 7 S1PR3^GRE-/GRE-^ mCherry  Post day 7 WT mCherry vs. post day 7 S1PR3^GRE-/GRE-^ mCherry  Post day 7 S1PR3^GRE-/GRE-^ hM4D vs. post day 7 S1PR3^GRE-/GRE-^ mCherry | Fisher’s LSD |  | <0.0001  <0.0001  <0.0001  <0.0001  <0.0001  <0.0001  0.0008  <0.0001  0.0002  <0.0001  <0.0001  0.0002  <0.0001  <0.0001  <0.0001 |
| 4i | Time interacting with stimulus rat | 0-5 min WT mCherry  0-5 min WT hM4D  0-5 min S1PR3^GRE-/GRE-^ mCherry  0-5 min S1PR3^GRE-/GRE-^ hM4D  5-10 min WT mCherry  5-10 min WT hM4D  5-10 min S1PR3^GRE-/GRE-^ mCherry  10-15 min S1PR3^GRE-/GRE-^ hM4D  10-15 min WT mCherry  10-15 min WT hM4D  10-15 min S1PR3^GRE-/GRE-^ mCherry  10-15 min S1PR3^GRE-/GRE-^ hM4D | 3-way ANOVA | Time F_1,38_ = 8.162  Genotype F_1,20_ = 25.74  DREADDs F_1,20_ = 65.68  Time x Genotype F_2,39_ = 21.819  Time x DREADDs F_2,39_ = 1.527  Genotype x DREADDs F_1,20_ = 0.8599  Time x Genotype x DREADDs F_2,39_ = 0.04301 | 0.0013  <0.0001  <0.0001  0.178  0.2302  0.3654  0.9579 |
|  |  | 0-5min:WT mCherry vs. 0-5min: S1PR3^GRE-/GRE-^ mCherry  0-5min:WT mCherry vs. 5-10min:WT hM4D  0-5min:WT hM4D vs. 0-5min: S1PR3^GRE-/GRE-^ mCherry  0-5min: S1PR3^GRE-/GRE-^ mCherry vs. 5-10min:WT mCherry  0-5min: S1PR3^GRE-/GRE-^ mCherry vs. 5-10min:WT hM4D  0-5min: S1PR3^GRE-/GRE-^ mCherry vs. 5-10min: S1PR3^GRE-/GRE-^ mCherry  0-5min: S1PR3^GRE-/GRE-^ mCherry vs. 10-15min:WT mCherry  0-5min: S1PR3^GRE-/GRE-^ mCherry vs. 10-15min:WT hM4D  0-5min: S1PR3^GRE-/GRE-^ mCherry vs. 10-15min: S1PR3^GRE-/GRE-^ hM4D  5-10min:WT mCherry vs. 5-10min:WT hM4D  5-10min:WT hM4D vs. 5-10min: S1PR3^GRE-/GRE-^ mCherry  5-10min:WT hM4D vs. 10-15min:WT mCherry  5-10min:WT hM4D vs. 10-15min: S1PR3^GRE-/GRE-^ mCherry  5-10min: S1PR3^GRE-/GRE-^ mCherry vs. 10-15min: WT hM4D  5-10min: S1PR3^GRE-/GRE-^ mCherry vs. 10-15min: S1PR3^GR-/GR^ hM4D  10-15 min: WT^-^ mCherry vs. 10-15min: WT^-^ hM4D  10-15min:WT mCherry vs. 10-15min: S1PR3^GRE-/GRE-^ hM4D  10-15min:WT hM4D vs. 10-15min: S1PR3^GRE-/GRE-^ mCherry  10-15min: S1PR3^GRE-/GRE-^ mCherry vs. 10-15min: S1PR3^GRE-/GRE-^ hM4D | Tukey’s multiple comparisons |  | 0.0033  0.0002  0.0248  0.0302  <0.0001  0.0132  0.0187  0.0063  0.0019  0.0270  0.0001  <0.0001  0.0032  0.0232  0.0164  0.0428  0.0381  0.0248  0.0473 |
| 4j | Mean LC-mPFC coherence across gamma frequency range (20-40 Hz) | WT female  S1PR3^GRE-/GRE-^ female | Two-way repeated measures ANOVA | Interaction F_51,459_ = 1.084  Frequency F_51,459_ = 3.95  Genotype F_51,459_ = 7.233 | 0.3285  <0.0001  0.0275 |
| 4k | Mean LC power spectral density percentage across gamma frequency range (20-40 Hz) | WT female  S1PR3^GRE-/GRE-^ female | Two-way repeated measures ANOVA | Interaction F_51,459_ = 1.131  Frequency F_51,459_ = 7.937  Genotype F_51,459_ = 5.283 | 0.2572  <0.0001  0.0471 |
